# Insights Into the Genetics of the Zhonghua 11 Resistance to *Meloidogyne graminicola* and Its Molecular Determinism in Rice

**DOI:** 10.3389/fpls.2022.854961

**Published:** 2022-05-04

**Authors:** Hue Thi Nguyen, Sophie Mantelin, Cuong Viet Ha, Mathias Lorieux, John T. Jones, Chung Duc Mai, Stéphane Bellafiore

**Affiliations:** ^1^LMI RICE-2, Agricultural Genetics Institute (AGI), Hanoi, Vietnam; ^2^Department of Life Sciences, University of Science and Technology of Hanoi (USTH), Hanoi, Vietnam; ^3^Institut National de Recherche pour l’Agriculture, l’Alimentation et l’Environnement (INRAE) UMR 1355 Institut Sophia Agrobiotech, Sophia Antipolis, France; ^4^Research Center of Tropical Plant Disease, Vietnam National University of Agriculture (VNUA), Hanoi, Vietnam; ^5^DIADE, University of Montpellier, IRD, CIRAD, Montpellier, France; ^6^The James Hutton Institute, Dundee, United Kingdom; ^7^School of Biology, University of St Andrews, St Andrews, United Kingdom; ^8^PHIM Plant Health Institute, University of Montpellier, IRD, CIRAD, INRAE, Institut Agro, Montpellier, France

**Keywords:** genetic determinism, hypersensitive response (HR), incompatible interaction, root-knot nematode (*Meloidogyne graminicola*), resistance, rice, salicylic acid

## Abstract

*Meloidogyne graminicola* is a widely spread nematode pest of rice that reduces crop yield up to 20% on average in Asia, with devastating consequences for local and global rice production. Due to the ban on many chemical nematicides and the recent changes in water management practices in rice agriculture, an even greater impact of *M. graminicola* can be expected in the future, stressing the demand for the development of new sustainable nematode management solutions. Recently, a source of resistance to *M. graminicola* was identified in the *Oryza sativa* japonica rice variety Zhonghua 11 (Zh11). In the present study, we examine the genetics of the Zh11 resistance to *M. graminicola* and provide new insights into its cellular and molecular mechanisms. The segregation of the resistance in F_2_ hybrid populations indicated that two dominant genes may be contributing to the resistance. The incompatible interaction of *M. graminicola* in Zh11 was distinguished by a lack of swelling of the root tips normally observed in compatible interactions. At the cellular level, the incompatible interaction was characterised by a rapid accumulation of reactive oxygen species in the vicinity of the nematodes, accompanied by extensive necrosis of neighbouring cells. The expression profiles of several genes involved in plant immunity were analysed at the early stages of infection during compatible (susceptible plant) and incompatible (resistant plant) interactions. Notably, the expression of *OsAtg4* and *OsAtg7*, significantly increased in roots of resistant plants in parallel with the cell death response, suggesting that autophagy is activated and may contribute to the resistance-mediated hypersensitive response. Similarly, transcriptional regulation of genes involved in hormonal pathways in Zh11 indicated that salicylate signalling may be important in the resistance response towards *M. graminicola*. Finally, the nature of the resistance to *M. graminicola* and the potential exploitation of the Zh11 resistance for breeding are discussed.

## Introduction

Plant parasitic nematodes (PPNs) are one of the most damaging pests in agriculture across all crops, and are estimated to cost up to $US 80 billion in losses per year worldwide (Nicol et al., 2011). The rice root-knot nematode (RKN), *Meloidogyne graminicola*, poses a serious threat to crop production in all rice agrosystems, while global rice consumption is forecast to rise (Mantelin et al., 2017; OECD/FAO, 2020). *M. graminicola* is a resilient pathogen. It is particularly well adapted to intermittent flooding and its eggs can persist in a dormant phase for at least 2–12 months in water-logged soil, before releasing infectious juveniles when infection conditions become favourable (Bridge and Page, 1982; Roy, 1982). In addition, *M. graminicola* has a wide host range, allowing it to find refuge in a large variety of rice-associated plants (Rich et al., 2009). Consequently, *M. graminicola* is persistent in infected fields, where it can reduce rice production by 20% on average and up to 80% in extreme infectious conditions (Netscher and Erlan, 1993; Soriano et al., 2000). Moreover, *M. graminicola*-infected plants are predisposed to other diseases due to weakened immunity (Kyndt et al., 2017), thus potentially leading to further indirect losses in rice production.

In order to reduce the impact of *M. graminicola* on rice agriculture, it is essential to develop integrated pest management systems that combine different approaches. Chemical nematicides have been widely used for decades with great efficiency before being withdrawn, in the vast majority of cases, due to their adverse effects on human health and the environment (Ebone et al., 2019). Alternative strategies such as flooding or crop rotation can be used, but they have serious limitations. On the one hand, continuous flooding of rice fields has an significant environmental cost due to production of methane (Redeker et al., 2000) and by increasing the constraints related to water management (De Waele and Elsen, 2007). On the other hand, crop rotations can efficiently lower nematode populations and improve rice yield (Soriano and Reversat, 2003; Win et al., 2019), but its implementation requires the culture of non-host crops, which is hampered by the wide host range of the nematode. Strategically, the genetic approach is a major component of most integrated pest management programs. Nematode-resistant varieties have been successfully deployed for PPN management in many crops (Barbary et al., 2015; Gartner et al., 2021) but this approach in rice has long been hampered by the lack of nematode resistance sources in the cultivated *Oryza sativa* species. Wild relatives such as *Oryza glumaepatula*, *Oryza longistaminata*, *Oryza nivara*, and *Oryza rufipogon* as well as African rice *Oryza glaberrima*, are known sources of genetic resistance to PPNs and to *M. graminicola* in particular (e.g., Soriano et al., 1999; Mattos et al., 2019; Hada et al., 2020). Although most of these are quantitative resistance sources, some of them have been successfully used in breeding for resistance to other PPNs (e.g., Lorieux et al., 2003) but not *M. graminicola*. More recently, studies of global rice panels have identified strong resistance to *M. graminicola* in Asian *O. sativa* varieties. Three of these resistances that originated from geographically and genetically diverse sources have been partially characterised: the Sri Lankan Indica variety LD 24 and the Thai *aus* subpopulation variety Khao Pahk Maw (KPM) (Dimkpa et al., 2016), as well as in the Chinese temperate japonica variety Zhonghua 11 (Zh11), the subject of the present study (Phan et al., 2018; Zhan et al., 2018).

At the start of the *M. graminicola*-rice compatible interaction leading to disease, *M. graminicola* stage 2 pre-infective juveniles (J2) hatched from eggs penetrate the root at the elongation zone close to the tip. After migrating intercellularly towards the root tip, the J2s invade the vascular cylinder and, unlike most other RKN species, establish their feeding site in the stele close to the root meristem instead of migrating upwards for a long distance (Kaloshian and Teixeira, 2019). Hypertrophy of the host cells surrounding the body of the nematode at the root tip contributes to the development of the distinctive terminal hook-shaped root knot in *M. graminicola* infected rice (Jena and Rao, 1977). The nematode induces the formation of its feeding site by reprogramming the host cells and then becomes sedentary. This feeding site is made of five to eight metabolically active giant cells that provide nutrients to the nematode for the rest of its development and sustain its reproduction (Cabasan et al., 2014). The *M. graminicola* life cycle takes about 20 days. During this time the adult female remains inside the roots, from where it releases several hundred eggs inside the cortex.

In an incompatible interaction that leads to resistance, plants may prevent the establishment of the nematode in the early stages of infection or later hamper the development and/or the maintenance of a functional feeding site. Several natural resistance (*R*) genes effective against *Meloidogyne* spp. have been reported from many plant species (Davies and Elling, 2015) but only two were cloned so far that have been validated: *Ma*, which confers broad-spectrum resistance to *Meloidogyne* spp. in Myrobalan plum, and *Mi-1.2*, which confers resistance to three species of RKN and to several phloem feeding insects in tomato (Milligan et al., 1998; Claverie et al., 2011; Kaloshian and Teixeira, 2019). *Mi-1.2* and *Ma* are both dominant resistance genes, belonging to the nucleotide-binding site (NBS) and leucine-rich repeat (LRR) receptor (NLR) family. Upon activation, both resistances trigger a hypersensitive reaction (HR), leading to a local cell-death that prevents the establishment of the nematode, causing its death most likely by starvation. Indeed, obligate sedentary endoparasites such as RKN must maintain a close biotrophic relationship with their host in order to survive but in *Mi-1.2* and *Ma*-mediated resistant plants, the progression of the infection is arrested before the nematode can initiate the formation of a proper feeding site (Melillo et al., 2006; Khallouk et al., 2011). These resistances partly rely on a sustained accumulation of reactive oxygen species (ROS) that is observed where HR also occurs. Furthermore, the defence response to *Meloidogyne* spp. mediated by *Mi-1.2* also depends on a complex signal transduction pathway in which phytohormones play a key role (Branch et al., 2004; Shukla et al., 2018; Kaloshian and Teixeira, 2019).

Similarly, resistance to *M. graminicola* in the African rice *O. glaberrima* involves a complex transcriptomic response where no specific hormonal pathway could be identified as the major determinant (Petitot et al., 2017). However, *M. graminicola* juveniles that penetrate the roots are persistent and able to induce the formation of giant cells. Histopathology studies have shown that in *M. graminicola*-resistant *O. glaberrima* lines the interaction may result in an HR-like response (Cabasan et al., 2014) but mostly the resistance is associated with a low level of nematode penetration and late giant cells degradation (Cabasan et al., 2012; Petitot et al., 2017). In contrast, *M. graminicola* infection in the resistant *O. sativa* Zh11 cultivar appears to trigger rapid cell death, with potentially necrotic cells observed in the root mesoderm during nematode migration, which also explains the complete inhibition of nematode multiplication and gall formation by *M. graminicola* (Phan et al., 2018). Nevertheless, the nature of the HR-like symptoms occurring during the *M. graminicola*-Zh11 rice incompatible interaction and the genetic determinants of the resistance remain to be identified.

The present study was aimed at deciphering the inheritance determinism of the resistance to *M. graminicola* in the *O. sativa* Zh11 rice and to elucidate at the cellular level the plant response during the incompatible interaction. To do this, we studied the segregation of the resistance in F_2_ hybrid populations issued from two different crosses, and complemented the genetic study with histological observations to precisely determine the spatio-temporal induction of the Zh11-mediated HR. We confirmed that the resistance to *M. graminicola* in Zh11 is dominant with seemingly two genes contributing to the resistance. In addition, we provide new insights into the plant molecular response to *M. graminicola* using comparative expression profiles of several genes involved in plant defence during both the compatible and the incompatible interactions in rice. Notably, two core plant autophagy genes were significantly upregulated early in the incompatible interaction, concomitant to the development of the HR, therefore suggesting that autophagy is activated and may contribute to the resistance-mediated hypersensitive response, as well as several hormonal pathways.

## Materials and Methods

### Rice Genotypes, Nematode Inoculation, and Growth Conditions

The rice variety Zhonghua 11 (*Oryza sativa* subsp. japonica) used in the present study was previously described as resistant to *M. graminicola* (Phan et al., 2018; Zhan et al., 2018) and was originally generated by another culture using a triparental cross: Jingfeng 5/Tetepu/Fujing (Lizhong Xiong, personal communication). The two rice varieties Nipponbare (*O. sativa* subsp. japonica) and IR64 (*O. sativa* subsp. indica) were used as susceptible varieties. Plants were grown as in lowland rice culture conditions, simulated by culture in hydroponics in a 1/4 Hoagland solution (Hoagland and Arnon, 1938). Briefly, rice seeds were dehulled and then pre-germinated for 7 days on autoclaved pure white silica sand (particle size: 0.1–0.4 mm) soaked to saturation with 1/4 of Hoagland’s solution before being transferred to small columns (5.5-cm-high, 3-cm-diameter), containing 16 g of autoclaved sand wetted with 1/4 Hoagland solution. Three days later (T0), plantlets were inoculated (or not, for nematode-free controls) with 100 J2 nematodes (or as otherwise stated) by pipetting the worm suspension around the stem’s base. At 4 days post-inoculation (dpi), plantlets were transferred to a 15-mL hydroponic culture system (Reversat and Fernandez, 2004) with Hoagland 1/4 solution. Plants were maintained in a culture room at 26–28°C (night-day), with a 12 h photoperiod (60 μmol.m^–2^.s^–1^ illumination) and at 78% relative humidity.

### Nematode Collection for Inoculation

*Meloidogyne graminicola* isolate Vn18 (Bellafiore et al., 2015) was maintained *in vivo* on the susceptible rice variety IR64 as described above. One month after infection, pre-parasitic J2 were collected from the hydroponic solution by sieving it through two layers of mesh, size 80 and 25 μm, consecutively. Juveniles were collected on the 25 μm mesh, rinsed several times with sterile double-distilled water (ddH_2_O), and placed on a 100 μm mesh stainless steel sieve draped with three layers of wet Kimwipe^®^ cloths over a 50-mL beaker filled with sterile ddH_2_O. After 24 h incubation in the dark at room temperature (ca. 28°C), the juveniles present in the beaker were used as inoculum.

### Crosses and Assessment of Resistance to *Meloidogyne graminicola* in the F_2_ Progenies

F_1_ hybrids were obtained by crossing the male donor resistant variety *O. sativa* japonica Zh11 with a susceptible female donor variety, either *O. sativa* indica IR64 (Phan et al., 2018) or *O. sativa* japonica Nipponbare (this study). F_1_ seeds were grown in the greenhouse in 5-L pots containing autoclaved soil. The hybrid identity of F_1_ plants was confirmed using simple-sequence repeats (SSR) markers (data not shown) and 12 of these were self-pollinated to obtain F_2_ seeds that were subsequently assessed for resistance. The F_2_ seeds from both crosses were planted and the 10-day-old F_2_ plants were then inoculated with 100 J2 (initial population; Pi) of *M. graminicola* Vn18 and grown in hydroponics in a culture room as described above. After 30 dpi, J2 nematodes were recovered from infected roots using the hypochlorite extraction method and a blender (McClure et al., 1973) with minor modifications (Bellafiore et al., 2015). The nematode suspension obtained after extraction was homogenised in tap water with volume adjusted to 50 mL and a 1 mL aliquot of this suspension was placed in a counting cell chamber to estimate the number of J2 produced after one cycle of reproduction (final population; Pf) using a stereomicroscope; counts made in triplicate were averaged for each plant. The reproductive factor (*Rf*) was calculated according to the ratio Pf/Pi. The screening of F_2_ progenies from the two crosses was performed independently, using the corresponding parents as control, either IR64 or Nipponbare as a susceptible control and Zh11 as a resistant control. A total of 242 F_2_ plants from the first cross (Zh11 × IR64) and 179 F_2_ plants from the second cross (Zh11 × Nipponbare) were evaluated. For each experiment, the maximum *Rf* value obtained for the resistant Zh11 parent plants was used as a reference value to define threshold for resistance in the screened F_2_ progenies, with plant scored as resistant for a *Rf* value inferior or equal to this threshold value. A Chi-square test was performed to measure the difference between the observed and expected phenotypes frequencies under a series of proposed segregation ratio hypothesis (Supplementary Tables 2, 3).

### 3,3-Diaminobenzidine Staining and Fresh Root Sectioning

DAB (3,3-diaminobenzidine) solution at 1 mg/mL (Sigma-Aldrich, Germany) was prepared as previously described (Daudi and O’Brien, 2012). Ten-day-old plants of Zh11 and IR64 were inoculated with 100 J2. At 2 dpi, roots were rinsed with water and immediately placed in the DAB solution in the dark and under vacuum for 1.5 h at room temperature (ca. 28°C). After staining, the roots were rinsed with water and longitudinal sections (25 μm-thick) were cut using a vibratome (Microm HM650V–Thermo Science) as previously described (Henry et al., 2016) with minor modifications: root tips (∼1 cm) were cut with a sharp blade and stored for 1 h in absolute ethanol before being embedded in 6% agarose blocks that were then sectioned with the vibratome on the same day. The sections were observed using a stereomicroscope (Olympus BX50, Japan) equipped with a Q-imaging digital camera either under UV light (UV A2 filter set, Zeiss AXIO Imager) or with standard brightfield optics after mounting the sections in 100% glycerol. The experiment was repeated three times with 10 plants for each time point. Representative samples are presented in Figure 2.

**FIGURE 1:**
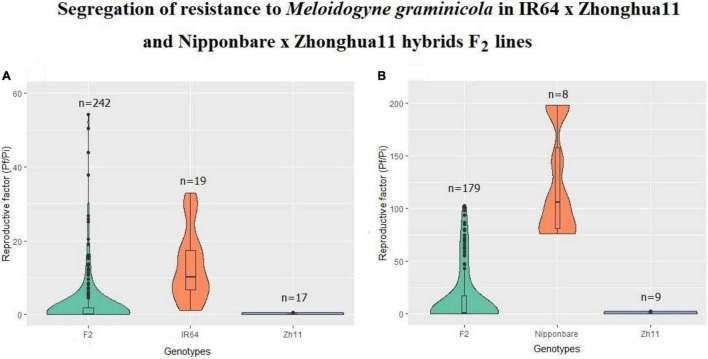
Reproduction factor of *Meloidogyne graminicola* on rice. Segregation of resistance observed in F_2_ progenies from crosses **(A)** IR64 × Zh11 and **(B)** Nipponbare × Zh11, compared to resistance level in Zhonghua 11 or susceptibility in susceptible parents, IR64 and Nipponbare, respectively. Reproduction factors (Pf/Pi) represents for each plant the number of nematodes recovered from roots (Pf) 30 days after inoculation of 100 *M. graminicola* stage 2 infective juveniles (J2).

**FIGURE 2:**
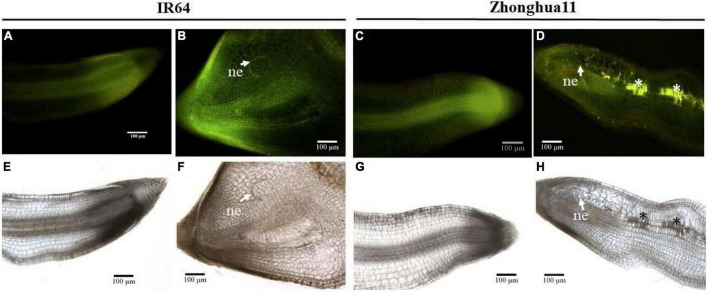
Reactive oxygen species accumulation in Zhonghua 11 roots was observed 2 days after *Meloidogyne graminicola* infection. Longitudinal root sections of healthy control and *M. graminicola*-inoculated roots of both susceptible IR64 and resistant Zh11 parents were observed at 2 dpi under UV light **(A–D)**, with glowing signal revealing the presence of phenolic compounds, or under white light **(E–H)** after DAB staining, with the formation of brown precipitates indicating accumulation of H_2_O_2_. **(A,E)** Non-infected IR64 roots; **(B,F)** Infected IR64 roots; **(C,G)** Non-infected Zh11 roots; **(D,H)** Infected Zh11 roots. Arrowheads indicate nematode (ne) juvenile in the root. Asterisks (*) show the ROS accumulation areas.

### Trypan Blue Staining

**FIGURE 3:**
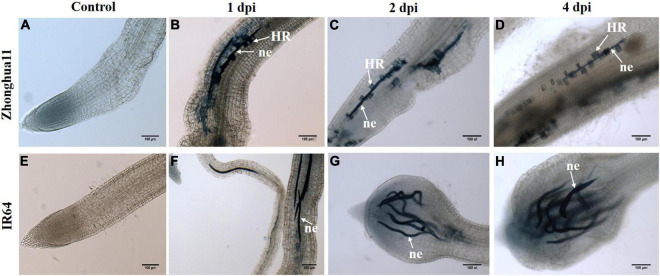
*Meloidogyne graminicola* triggers plant cell death in Zhonghua 11 with dead cells paving the way along the nematode migration tracks in roots. Trypan blue staining of roots in **(A–D)** Zhonghua 11 and **(E–H)** IR64 plant before (control) and after infection with *M. graminicola* at 1, 2, and 4 dpi, respectively. ne, nematode; HR, hypersensitive reaction. Both the nematode juveniles and the root dead cells appear in blue colour. Scale bar: 100 μm.

Ten-day-old plants of Zh11 and IR64 were inoculated with 360 juveniles. At 1, 2, and 4 dpi, roots were sampled and washed carefully with water to remove sand, then soaked for 7 min in 0.5% sodium hypochlorite (NaOCl) before being rinsed with distilled water for 10 min. Roots were then stained with 0.1% Trypan blue (Sigma-Aldrich, Germany) in a boiled solution of distilled water: lactic acid: glycerol: phenol (ratio 1:1:1:1). The roots were finally bleached in a boiling solution of 2.5 g/mL chloral hydrate for 1 min, and transferred to fresh chloral hydrate solution at room temperature overnight. Fragments of 1 cm root from the tip were excised and mounted on a glass slide in a drop of 100% glycerol. The whole mounted roots were then observed using a stereomicroscope (Olympus BX50, Japan) equipped with a Q-imaging digital camera with standard brightfield optics. Three independent experiments were performed with 10 plants at each time point. Representative samples are presented in Figure 3.

### Analysis of Gene Expression by Quantitative Reverse-Transcription PCR

For gene transcription analysis, resistant Zh11 and susceptible IR64 parental genotypes were used. *M. graminicola* infected (inoculation with 100 J2) and nematode-free control roots were collected at 1, 2, and 4 dpi. Using a stereomicroscope and a razor blade, 10 root tips (ca. 1 cm long) per sample were cut, pooled, and immediately frozen in liquid nitrogen. Samples were then stored at −80°C until used for RNA extraction. Total RNA for each sample was extracted using the RNeasy Plant Mini Kit (Qiagen, Germany), including a DNase treatment on column, according to the manufacturer’s guidelines, and 500 ng of total RNA for each sample was reverse-transcribed using the SuperScript III kit and oligo (dT) primers according to the manufacturer’s recommendations (Invitrogen/Thermo Fisher Scientific, United States). The targeted genes were PCR-amplified with a qPCR Mx3005P (Agilent Technology, United States) in a 10-μL reaction mix, containing 10 ng of cDNA, 300 nM of each gene-specific primer (Supplementary Table 1), and 1X SYBR Green GoTaq qPCR buffer (Promega, United States), with the following program: 5 min at 95°C and 40 cycles of (15 s at 95°C, 20 s at 58°C, and 30 s at 72°C). Three biological replicates consisting of ten plants for each combination (genotype × infection condition × time point) were used for the experiment. Each reaction was technically performed in triplicate and the *OseEF-1A* gene (*eukaryotic translation Elongation Factor 1A*; Os03g0177400) was used as housekeeping gene for normalisation (Nguyễn et al., 2014). Relative gene expression was calculated using the 2^–ΔΔCT^ method, comparing nematode-inoculated roots to uninfected roots sampled at the same time points (Livak and Schmittgen, 2001) and are presented as the mean of log2 ± SE. Significant variations between the gene expression in root inoculated vs. non-inoculated conditions was assessed using the Student’s *t*-test at each time point.

## Results

### Resistance Segregation in F_2_ Hybrids Revealed an Oligogenic Determinism of Zhonghua 11 Resistance to *Meloidogyne graminicola*

In order to assess whether the inheritance of the Zh11 resistance to *M. graminicola* was either simple (based on a single gene) or complex (relying on several interacting genes, and potentially involving epistasis phenomena), resistance phenotyping was carried out on F_2_ hybrid populations. The F_2_ lines were obtained from self-fertilisation of F_1_ individuals originating either from an inter-subspecific cross (*O. sativa* indica var. IR64 × *O. sativa* japonica var. Zh11) or from an intra-subspecific *O. sativa* japonica cross (Nipponbare × Zh11). The F_2_ plants from both crosses were inoculated with the same *M. graminicola* population under similar conditions and the segregation of the resistance was evaluated based on the level of nematode infection compared to the response observed in the susceptible parents (IR64 or Nipponbare) and in the resistant parent Zh11. The reproduction factor (*Rf*) of *M. graminicola* for each plant was calculated after one complete reproduction cycle [30 days post-infection (dpi) with *M. graminicola*]. As nematode infection rate is also dependent on the physiological state of both the plants and the inoculum (pre-parasitic J2), the threshold for determining resistance was not empirically fixed at *Rf* < 1 but defined from a set of control plants in each resistance assessment experiment.

In the original indica × japonica cross (IR64 × Zh11), the F_1_ plants were statistically as resistant as the Zh11 “male donor parent” (Phan et al., 2018). In the F_2_ resistance assessment assay (Figure 1A), the average *Rf* (±SD) of *M. graminicola* on IR64 and Zh11 plants were 13.22 ± 9.52 (ranging from 1.2 to 32.86; *n* = 19) and 0.04 ± 0.16 (ranging from 0 to 0.64; *n* = 17), respectively. The maximum *Rf* observed in Zh11 parental donor, *Rf* = 0.64, was used as a cut-off value to determine the threshold below which F_2_ progenies were considered resistant. Likewise, F_2_ plants were considered susceptible with a *Rf* > 0.64. The resistant to susceptible (R:S) segregation ratio was then calculated based on the full dataset (with *Rf* ranging from 0 to 54.3 across 242 F_2_ plants; Figure 1A), as well as after removing individuals falling in a 5 or 10% interval around the 0.64 *Rf* value, which did not change the final statistical outcome (Supplementary Table 2). The resistance segregation observed for the F_2_ plant population (Table 1; IR64 × Zh11) deviated significantly from the ratios of 3:1 (χ^2^ = 16.67, *p* = 0.00006), 15:1 (χ^2^ = 374.53, *p* < 0.00001) and 63:1 (χ^2^ = 1,905.56, *p* < 0.00001), which would have been expected for a resistance depending on either one, two or three independent dominant *R* genes. The Chi-square test analysis instead supported that the resistance in F_2_ follows a segregation based on a 11:5 ratio (χ^2^ = 2.95, *p* = 0.089) (Table 1), which would imply that two dominant genes may be contributing to the resistance, with a complete resistance achieved only if one dominant resistant allele is present for each of the two genes.

**TABLE 1:** Segregation ratios for resistance to *Meloidogyne graminicola* in F_2_ populations from two different crosses.

IR64 × Zh11	Total no. of F_2_	Tested ratio	DF	Observed		Test of statistics	
	242			R (Rf ≤ 0.64)	S (Rf > 0.64)	X^2^	Probability (*p*)
				154	88		
		3:1	1			16.67	0.00006
		1:3	1			192.67	<0.00001
		9:7	1			5.37	0.02
		7:9	1			38.89	<0.00001
		5:11	1			118.15	<0.00001
		11:5	1			2.95	0.089*
		13:3	1			49.28	<0.00001
		3:13	1			320.05	<0.00001
		15:1	1			374.53	<0.00001
		1:15	1			1,360.13	<0.00001
		63:1	1			1,905.56	<0.00001
		1:63	1			6,062.51	<0.00001

**Nipponbare × Zh11**	**Total no. of F_2_**	**Tested ratio**	**DF**	**Observed**		**Test of statistics**	
	**179**			**R (Rf ≤ 2.5)**	**S (Rf > 2.5)**	**X^2^**	**Probability (*p*)**
				**98**	**81**		

		3:1	1			39.15	<0.00001
		1:3	1			84.49	<0.00001
		9:7	1			0.16	0.7*
		7:9	1			8.80	0.0033
		5:11	1			46.01	<0.00001
		11:5	1			16.33	0.00004
		13:3	1			82.52	<0.00001
		3:13	1			152.26	<0.00001
		15:1	1			464.69	<0.00001
		1:15	1			718.55	<0.00001
		63:1	1			2,221.34	<0.00001
		1:63	1			3,292.07	<0.00001

*corresponds to the most significant ratio of resistance segregation.

In the Nipponbare × Zh11 cross, all the phenotyped F_1_ plants were resistant, confirming that the resistance to *M. graminicola* in Zh11 is dominant (data not shown). In the F_2_ resistance assessment assay (Figure 1B), the average *Rf* (±SD) of *M. graminicola* on Nipponbare and Zh11 plants were 123.30 ± 50.69 (ranging from 75.90 to 198; *n* = 8) and 0.50 ± 0.90 (ranging from 0 to 2.50; *n* = 9), respectively. The maximum *Rf* observed in Zh11 parental donor, *Rf* = 2.50, was used as a cut-off value to determine the threshold below which F_2_ progenies were considered resistant. Likewise, F_2_ plants were considered susceptible with a *Rf* > 2.50. The R:S segregation ratio was then calculated based on the full dataset (with *Rf* ranging from 0 to 102.60 across 179 F_2_ plants; Figure 1B), as well as after removing individuals falling in a 5 or 10% interval around the 2.50 *Rf* value, which did not change the final statistical outcome (Supplementary Table 3). The resistance showed a 9:7 segregation ratio (χ^2^ = 0.16, *p* = 0.70; Table 1), indicating that two complementary dominant genes most-likely contribute to the observed phenotype, thus confirming the hypothesis based on the analysis of the segregation from the inter-subspecific cross.

### *Meloidogyne graminicola*-Triggered Incompatible Interaction in Zhonghua 11 Is Associated With Accumulation of Reactive Oxygen Species

In most cases the *R*-mediated incompatible response leads to a HR and *M. graminicola* infection appears to trigger rapid cell death in Zh11 (Phan et al., 2018). Bursts of ROS and NO usually occur early upon pathogen recognition in plants and are often important for the initiation of the HR (Delledonne et al., 2001), with the ROS acting as a signalling molecule to activate the plant’s defences (Bhattacharjee, 2012). To determine whether the low *M. graminicola Rf* observed in the Zh11 parent is due to an active plant defence response, the production of ROS was investigated in nematode-infected root tissues. DAB (3,3′-diaminobenzidine) staining at an early stage of infection (2 dpi) was used to observe accumulation of ROS in rice roots, based on the formation of brown precipitates visible under white light upon reaction between DAB and hydrogen peroxide (H_2_O_2_). At this early time of infection, *M. graminicola* was already initiating feeding sites in the IR64 compatible parental host, as evidenced by the swelling of the root tips, and without visibly triggering a ROS burst or an accumulation of aromatic compounds (Figures 2B,F). By contrast, infected roots of the incompatible Zh11 parent did not show any effect of hypertrophy and hyperplasia of the root cells, and brown spots were visible along *M. graminicola* migration tracks, indicating accumulation of ROS in response to the nematode infection (Figure 2H). ROS-accumulating root cells in Zh11 also showed concomitant accumulation of aromatic compounds, revealed by the intense yellow/orange autofluorescence of the cells observed under UV light (Figure 2D; Pegard et al., 2005).

## *Meloidogyne graminicola*-Triggered Resistance in Zhonghua 11 Is Characterised by a Rapid Local Cell Death

It was established that ROS accumulates in Zh11 roots in response to *M. graminicola* infection, yet a ROS burst marks the onset of a typical HR response that in most cases occurs in plants during the incompatible interaction (Balint-Kurti, 2019). The HR is a form of programmed cell death localised at the site of pathogen infection. In the initial histopathology study of the Zh11 resistance, toluidine blue staining evidenced the formation of apoptotic cells surrounding the nematode in roots upon *M. graminicola* infection (Phan et al., 2018). In order to confirm the nature of this cellular response, *M. graminicola*-infected roots were stained with trypan blue to identify cell death; while living cells can effectively exclude the dye, dead cells with compromised membrane integrity become blue. One day after infection, nematodes (worms stained in blue) had penetrated the roots in both resistant Zh11 and susceptible IR64 plants and migrated towards the vascular cylinder (Figure 3). In Zh11, trypan blue-stained dead cells were observed along the nematode migration tracks in roots from 1 to 4 dpi. By contrast, at 2 and 4 dpi, hypertrophy and hyperplasia at the root tip were already pronounced in IR64 in response to the infection, and a lack of trypan blue cell staining in the roots was noticeable (Figures 3G,H). No staining was observed in non-inoculated control roots for either rice genotypes (Figures 3A,E). These data indicate that cell death was activated in Zh11 from the early stage of nematode infection, and that consequently HR-mediated cell death may be one of the determinants of the observed incompatible interaction in Zh11 rice in response to *M. graminicola*.

### *Meloidogyne graminicola*-Mediated Hypersensitive Response in Zhonghua 11 Is Associated With Changes in the Expression of Genes Involved in Autophagy

**FIGURE 4:**
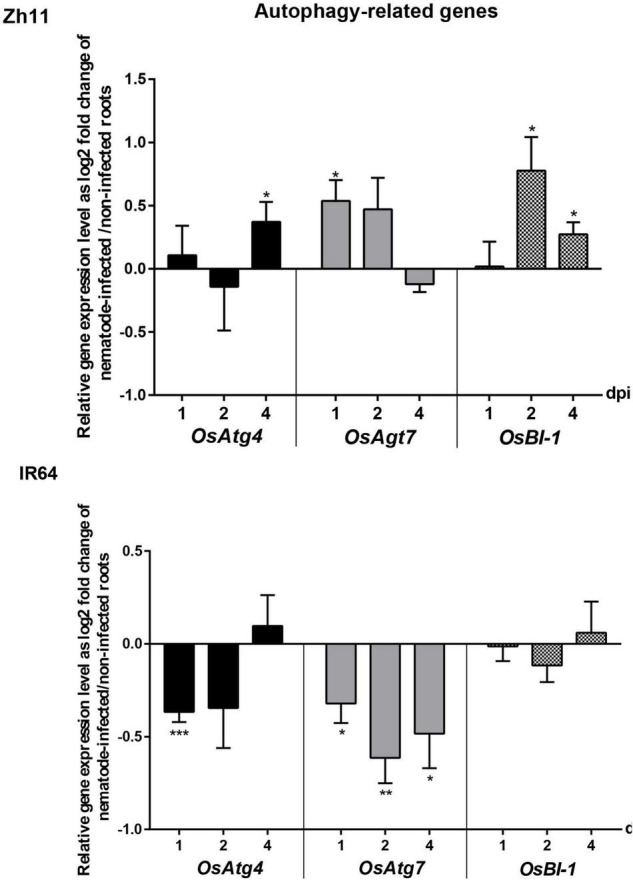
Temporal expression pattern of genes involved in the autophagy process during the incompatible interaction in Zh11 and compatible interaction in IR64 with *Meloidogyne graminicola*. The gene expression patterns are presented in parallel for Zh11 and IR64. Ten root tips per sample were collected and pooled for inoculated and non-inoculated plants at 1, 2, and 4 days post nematode infection (dpi). Gene expression levels were measured by qRT-PCR in triplicates, for three independent biological repeats for each combination (genotype/treatment/time point) and normalised using the internal reference gene *OseEF-1A* (Os03g0177400). Bars represent at each time point the relative gene expression as log2 fold changes of the mean and standard error for nematode-inoculated root sample compared with uninfected control plants grown under the same conditions The result of the Student’s *t*-test comparing for each time point the gene expression in root inoculated vs. non-inoculated conditions is indicated above the bar when there is a significant difference of the means with *p* value < 0.05 (*), *p* ≤ 0.01 (**), or *p* ≤ 0.001 (***). Genes investigated: autophagy-related genes *OsAtg4* (Os04g0682000) and *OsAtg7* (Os01g0614900); BAX inhibitor *OsBI-1* (Os02g0125300).

Since resistance in Zh11 is characterised by a rapid and localised HR, we investigated the putative involvement of autophagy in the response. Autophagy is indeed necessary for the proper regulation of *R*-mediated HR but also a complex process involving many regulatory and core genes (Wang et al., 2021). Three genes known to be transcriptionally regulated upon activation of autophagy in plants and present as a single copy in rice were selected as markers (*OsAtg4, OsAtg7*, and *OsBI-1*). Their transcriptional regulation patterns were analysed at early stages of infection (1, 2, and 4 dpi) in the parental genotypes Zh11 and IR64 by quantitative real-time polymerase chain reaction (qRT-PCR; Figure 4). In Zh11, both core autophagy genes *OsAtg4* and *OsAtg7* were significantly upregulated in *M. graminicola*-infected roots, respectively, at 4 and 1 dpi, concomitantly to the onset of HR development (Figures 3B,C). By contrast, those two genes were significantly downregulated in the IR64-*M. graminicola* compatible interaction. In addition, the cell death regulator *OsBI-1* that positively regulates autophagy showed constant expression in IR64, while in Zh11 *OsBI-1* was significantly and transiently upregulated upon nematode infection at 2 and 4 dpi (Figure 4). Altogether these results suggest that autophagy may be involved in the HR-mediated cell death leading to resistance in Zh11, while it might be repressed in compatible interaction during the early stages of infection.

### Insights Into Early Expression of Genes of Hormone-Related Pathways Classically Involved in Rice Response to Biotic Stresses

**FIGURE 5:**
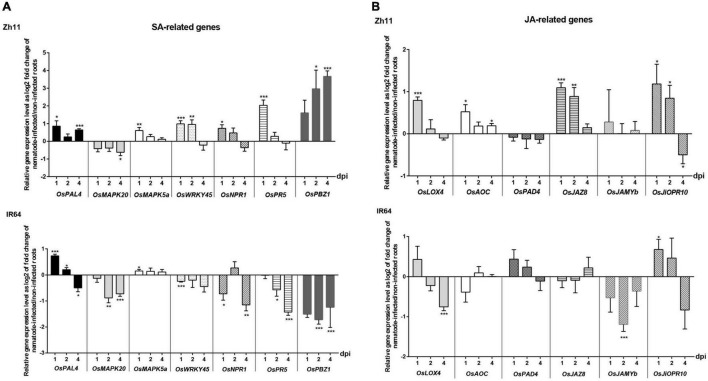
Temporal expression pattern of genes involved in the salicylic acid (SA) and jasmonate (JA) hormonal pathways during the incompatible interaction in Zh11 and compatible interaction in IR64 with *Meloidogyne graminicola*. **(A)** Expression level of genes involved in the hormone synthesis, signalling, or response to SA. **(B)** Expression level of genes involved in the hormone synthesis, signalling, or response to JA. In each panel the gene expression patterns are presented in parallel for Zh11 and IR64. Ten root tips per sample were collected and pooled for inoculated and non-inoculated plants at 1, 2, and 4 days post nematode infection (dpi). Gene expression levels were measured by qRT-PCR in technical triplicates, for three independent biological repeats for each combination (genotype/treatment/time point) and normalised using the internal reference gene *OseEF-1A* (Os03g0177400). Bars represent at each time point the relative gene expression as log2 fold changes of the mean and standard error for nematode-inoculated root sample compared with uninfected control plants grown under the same conditions. The result of the Student’s *t*-test comparing for each time point the gene expression in root inoculated vs. non-inoculated conditions is indicated on top of the bar when there is a significant difference of the means with *p* value < 0.05 (*), *p* ≤ 0.01 (**), or *p* ≤ 0.001 (***). Genes investigated: the phenylalanine ammonia-lyase *OsPAL4* (Os02g0627100); the phytoalexin deficient *OsPAD4* (Os11g0195500); the mitogen-activated protein kinases *OsMAPK20* (Os01g0629900) and *OsMAPK5a* (Os03g0285800); the WRKY transcription factor *OsWRKY45* (Os05g0322900); the non-expressor of pathogenesis-related genes *OsNPR1* (Os01g0194300); the pathogenesis-related *OsPR5* (Os12g0628600) and *OsPR10a/PBZ1* (Os12g0555500); the lipoxygenase *OsLOX4* (Os03g0700400); the allene oxide cyclase *OsAOC* (Os03g0438100); the jasmonate-ZIM-domain 8 *OsJAZ8* (Os09g0439200); the R2R3 MYB *OsJAMYb* (Os11g0684000); the jasmonic acid inducible pathogenesis-related class 10 *OsJiOPR10* (Os03g0300400).

The resistance response elicited in Zh11 by *M. graminicola* infection leads to a HR, with autophagy most likely involved. There is mounting evidence that hormone signalling plays critical roles in regulating autophagy and in plant stress responses in general, although integration of the signals and how hormones cross-talk are still poorly understood (Gou et al., 2019; Liao and Bassham, 2020). Nevertheless, unlike in dicots, synergistic SA–JA interactions seem to take place in rice defence response to pathogens (De Vleesschauwer et al., 2014). To get insight into these two major plant hormonal pathways that may be responding to nematode infection in the *M. graminicola*-Zh11 incompatible interaction, we analysed the expression level of several genes involved in the biosynthesis and/or the signalling of SA and JA at early stages of infection (1, 2, and 4 dpi) by qRT-PCR (Figure 5).

Overall, there was an opposite response of Zh11 and IR64 in regards to the SA-related pathways (Figure 5A) upon *M. graminicola* infection. During the incompatible interaction in Zh11, the hormone biosynthetic gene *OsPAL4* was slightly but significantly upregulated. In addition, most SA-marker genes were upregulated (*OsMAPK5a*, *OsWRKY45*, *OsNPR1*, *OsNPR5*, and *OsPBZ1*), with a noticeable sustained induction of the plant cell-death inducer *OsPBZ1* (Kim et al., 2011) that was significant at 2 and 4 dpi. By contrast, there was a consistent and significant down regulation of most SA-signalling and responsive marker genes (all except *OsMAPK5*) at the different time points after infection in the susceptible IR64 despite a transient upregulation of *OsPAL4* at the earliest time points.

The results observed for the JA-related gene responses (Figure 5B) were overall less significant than for SA. Remarkably, JA biosynthetic genes, *OsLOX4* and *OsAOC*, were upregulated in infected roots of Zh11, while they were either downregulated or not significantly affected in response to the nematode infection in the susceptible IR64 rice. The significant upregulation of the JA-signalling repressor *OsJAZ8* (Yamada et al., 2012) that was specifically observed in Zh11 infected roots at 1 and 2 dpi was, however, consistent with the putative production of JA. It may also explain the lack of response in other JA-related genes such as *OsPAD4* or *OsJAMYb*.

Taken together these gene expression profiling analyses highlighted a putative role for SA rather than JA in resistance to *M. graminicola* in Zh11.

## Discussion

*Meloidogyne graminicola* resistance in rice is a complex trait that seems to require a number of genes that have not yet been clearly identified but are distributed on different chromosomes (Hada et al., 2020). Natural resistance to *M. graminicola* was first reported in *O. longistaminata* and *O. glaberrima* (Soriano et al., 1999), suggesting that rice germplasm may carry important resistance genes, useful for breeding elite rice varieties. These genes could thus be exploited to support sustainable rice production where the use of environmentally toxic nematicides has been banned. Efforts have been made to introgress *M. graminicola* resistance from *O. glaberrima* to *O. sativa*. However, interspecific progenies never reached the same degree of resistance as in the *O. glaberrima* donor parents (Plowright et al., 1999; Bimpong et al., 2010). More recently, several studies have been undertaken aiming at identifying resistant varieties in wild rice and in the commonly grown and consumed Asian rice *O. sativa*. Remarkably, three varieties of *O. sativa* have recently been identified that showed effective resistance to *M. graminicola* (Dimkpa et al., 2016; Phan et al., 2018) and that represent new sources of resistance for breeders, allowing direct introgression into *O. sativa* elite varieties. Nevertheless, prior knowledge of the genetic determinism and resistance-mediated defence mechanisms observed in these resistant varieties is needed before considering their use in breeding programmes and eventually pyramiding the resistance sources.

The aim of this study was to decipher the genetic inheritance of the resistance to *M. graminicola* in Zh11 and undertake the characterisation of its mode of action at the cellular and molecular level. We found that (*i*) the defence response in Zh11 is characterised by an HR, with autophagy potentially involved in the HR-mediated cell death; and (*ii*) the resistance in Zh11 is associated with transcriptional reprogramming of defence-related genes at early stages of infection, with induction of the SA-signalling pathway; (*iii*) the resistance to *M. graminicola* in Zh11 is most likely controlled by two genetically unlinked dominant and complementary genes.

### Hypersensitive Response-Mediated Cell Death Is a Characteristic Determinant of the Execution of the Resistance to *Meloidogyne graminicola* in Zhonghua 11 That May Be Promoted by Autophagy

The plant-pathogen incompatible reaction relies on the activity of resistance genes to inhibit or prevent the establishment of the biotrophic pathogens and restrain the progression of the disease. The resistance is achieved through a series of events that ultimately lead, in most cases, to a local and tightly controlled plant programmed cell death. This incompatible reaction is therefore marked at the site of infection by the presence of necrotic tissues resulting from several concomitant events elicited by the pathogen and controlled by the plant, such as ROS production, lipid peroxidation, loss of enzymatic activity, and autophagy. These changes lead to the HR, which is one of the main manifestations of *R*-gene-mediated resistance, even though resistance and HR are unlinked in some situations that, however, remain the exception (Künstler et al., 2016). A burst of ROS production usually occurs early in the plant defence response and plays a key role in the orchestration of the HR (Delledonne et al., 2001; Balint-Kurti, 2019). The oxidative burst involves reactive oxygen intermediates such as the free radical superoxide anion (O^2–^), H_2_O_2_ and the hydroxyl radical (OH^–^), which have a strong oxidative activity. Their abundance in the plant cells is an early marker of HR and quickly leads to cell death (Delledonne et al., 2001). Typically, a sustained accumulation of H_2_O_2_ is observed in the *Mi-1.2* resistant tomato in response to the root-knot nematode *Meloidogyne incognita*. In addition, it was notably demonstrated that the timing of H_2_O_2_ generation is determinant in blocking the nematode establishment (Melillo et al., 2006). Nevertheless, ROS may also be produced in susceptible plants under nematode attack, that participate in the plant basal defence in response to nematode elicitors (e.g., ascarosides) or damage-associated molecules produced during the migration of the nematode inside the roots (Choi and Klessig, 2016; Sato et al., 2019).

In the present study, we show that ROS in the form of H_2_O_2_ accumulated rapidly after *M. graminicola* infection and in significant quantity that could be detected after only 1 dpi in Zh11 incompatible host. This is the sign that the plant had quickly detected the pathogen while it was still migrating through the root, most likely when it was probing the cells along its way to find one suitable cell to establish its feeding site, based on the observed pattern of ROS staining in the roots. By contrast, no ROS accumulation was observed in DAB-stained roots after 1–4 days post *M. graminicola* infection in compatible hosts, while the roots underwent obvious changes related to nematode establishment, such as a characteristic hypertrophy and hyperplasia of the root tip. Anti-oxidant enzymes, such as superoxide dismutase (SOD), thioredoxin, and glutathione peroxidase, are secreted by RKNs (Bellafiore et al., 2008). It has been hypothesised that these enzymes protect the nematodes from host-induced oxidative damage (Callahan et al., 1988). Similarly, two protein disulphide isomerases (PDI) secreted by *M. graminicola* have been characterised that can protect *M. graminicola* from the ROS released by rice in compatible interaction (Tian et al., 2019, 2020). These secreted enzymes could locally detoxify the nematode environment at the site of infection and allow the parasite to establish its feeding site, like we observed in the susceptible IR64 rice after *M. graminicola* infection. However, during the incompatible interaction with Zh11, the massive production of reactive oxygen intermediates did not allow the activity of these detoxifying enzymes to thwart the oxidative burst and prevent the HR. Not only could ROS be detected in the cells bordering the *M. graminicola* migration sites in Zh11, but some of these cells also initiated a cell death process as shown by trypan blue staining. Over the past decade, autophagy has emerged as a key regulator of plant innate immunity. It is a catabolic self-degradative process that promotes pathogen-induced hypersensitive cell death but restricts unnecessary spread of cell death (Leary et al., 2018; Zeng et al., 2019). For instance, autophagy participates in *Xanthomonas oryzae* pv. *oryzae* (*Xoo*) resistance mediated by *Xa3/Xa26* in rice (Cao et al., 2019). The hypersensitive response programmed cell death induced by the bacteria during incompatible interaction was associated with transient upregulation in the leaf of several autophagy-related genes in the resistant plants, including *OsAtg7*, shortly after inoculation of the pathogen. Similarly, *OsAtg7* was markedly upregulated in different rice lines harbouring dominant resistance genes that lead to cell death upon inoculation with *Xoo* (Cao et al., 2018). In addition, *OsAtg4* was used as a marker of autophagy in rice to demonstrate the activation of this process in cell death induced by the *Magnaporthe oryzae*-Secreted Protein 1 (MSP1; Wang et al., 2016). In the present study we demonstrated that two of the core autophagy genes, *OsAtg4* and *OsAtg7*, were specifically and transiently upregulated in response to *M. graminicola* infection in the root during incompatible interaction in Zh11. Moreover, the gene encoding the cell death regulator that positively regulates autophagy, *OsBI-1* (Xu et al., 2017), was transiently upregulated in the same plants. This transcriptomic regulation occurred concomitantly to the onset of HR development in the roots infected with *M. graminicola*. It is therefore likely that autophagy was involved in the HR-mediated cell death leading to resistance in Zh11.

The HR can occur at different stages of infection and this kinetic largely depends on the nature of the resistance, which leads to a response specific to each pathosystem. For example, two types of *R*-gene-mediated HR have been described in pepper: *Me3* (in PM687) controls an HR at the early stage of infection, surrounding the nematode entry site in the root epidermis in response to *Meloidogyne incognita*, and *Me1* (in PM217) acts at a later stage of infection, with *Me1*-containing plants developing a more progressive resistance reaction to *Meloidogyne incognita*, about 3 days later than *Me3*-containing plants, when the nematode is in the inner cortex near the vascular cylinder; these kinetic responses were the exact opposite in the same plants inoculated with *M. arenaria* (Hendy et al., 1985). In rice, *M. graminicola* penetrates the roots of susceptible (IR64) and resistant (Zh11) plants at similar levels (Phan et al., 2018). However, necrosis occurred immediately after invasion in the resistant Zh11 plants, with some root cells bordering the migration site showing signs of cell death as early as 1 dpi. This is very different from the observed histopathology in the African rice *O. glaberrima* (Cabasan et al., 2014; Petitot et al., 2017), where the resistance is mostly associated with low level of nematode penetration (40% fewer nematodes penetrated the TOG5681 at 1 dpi compared with susceptible Nipponbare roots, and overall, 80% less nematodes were found in the resistant plants after 7 dpi) and late giant cells degradation. Since *M. graminicola* is rapidly detected in Zh11, it is possible to speculate about the factor(s) produced by the nematode that trigger the resistance. PPNs secrete pathogenicity effectors, essentially proteins, into the apoplast and/or the cytoplasm of the probed host root cells, some of which are involved in plant immune recognition and signalling (Sato et al., 2019). In resistant plants, the incompatible interaction often relies on the detection of some of these effectors, which are recognised as avirulence factors by intracellular nucleotide-binding domain leucine-rich repeat (NLR)-type of immune sensors, resistance proteins, and that results in NLR-triggered immunity (van Wersch et al., 2020). In plant-PPN incompatible interactions, identified resistance loci mostly encode NLRs, even though only one cognate avirulence factor has ever been identified so far (Sato et al., 2019). *M. graminicola* infective juveniles express a myriad of effectors before and during interaction in rice (Petitot et al., 2020). We cannot conclude whether the root cells in Zh11 triggered the HR due to the detection of secreted effector(s). However, the observation of a rapid HR after infection and the almost total absence of reproduction of *M. graminicola* in Zh11 suggests that an R/Avr interaction maybe initiating the incompatible response and that one or more NLR proteins could be involved in the immune response.

### *Meloidogyne graminicola* Infection Induce Specific Changes in Temporal Expression of Genes Related to the Plant Defence Hormones in Zhonghua 11

In plant-nematode compatible interactions, phytohormones show a complex interplay between defence signalling and development (Gheysen and Mitchum, 2019) because the nematode needs to recruit specific hormone-dependent plant developmental pathways to establish its feeding site and at the same time needs to suppress plant defences, which also requires manipulation of phytohormone pathways (Thaler et al., 2012; Cohen and Leach, 2019; Yang et al., 2019). Following infection by *M. graminicola* in compatible interactions in rice, profound changes in plant gene expression occur (Kyndt et al., 2012; Ji et al., 2013; Petitot et al., 2017). These transcriptomic studies highlighted in particular that the compatible *M. graminicola*-rice interaction is tightly regulated under phytohormones control, with JA playing a central role in basal defence (Kyndt et al., 2012; Ji et al., 2013), while SA and ethylene only had a minor defence-inducing capacity (Nahar et al., 2011). In these analyses, the authors point out that transcriptomic regulation of genes related to phytohormone metabolism and signal transduction pathways is an important component of the plant response featuring compatible rice-*M. graminicola* interactions. In addition, it has been shown that during compatible interaction, the rice defence against RKN is attenuated and that the SA local defence in particular is suppressed (Kyndt et al., 2013). Similarly, we have shown that the rice defence to *M. graminicola* in the susceptible IR64 might be attenuated, with a consistent and significant down regulation of many SA-signalling and responsive genes (Figure 5A). However, the JA-related response was not particularly strong in IR64 and could have be damped by the nematode that induced a downregulation of the JA synthesis gene *OsLOX4*, as well as a downregulation of the JA-dependent MYB transcription factor *OsJAMYb* (Lee et al., 2001).

In the present study, we have also shown that the resistance to *M. graminicola* in Zh11 triggers cell death. Our results suggest that this reaction could be SA-dependent, with a strong upregulation of the SA-responsive plant cell-death inducer *OsPBZ1* (Kim et al., 2011). Concomitant to the hypersensitive reaction observed that is triggered by the nematode infection in Zh11, the resistant plant immediately activates a SA-dependent defence response, which is visible at the molecular level directly after penetration. The expression of *OsPAL4*, which is involved in the biosynthesis of SA, was slightly by significantly upregulated at 1 dpi during the incompatible interaction. However, endogenous SA level is known to be high in rice cells (Yang et al., 2014), so the hormonal response to the nematode infection would be occurring mainly in the SA signalling pathway. Accordingly, the expression of the pathogenesis-related gene *OsPR5*, known to be induced by SA and associated with systemic acquired resistance (SAR) in rice (Ganesan and Thomas, 2001), was significantly upregulated in Zh11 in response to *M. graminicola* infection at 1 dpi. The SA signalling pathway is dichotomic in rice, with the WRKY45-dependent and the NH1/OsNPR1-dependent pathways (Takatsuji et al., 2010; Ueno et al., 2015). These two key genes, *OsWRKY45* and *OsNPR1*, orchestrate the integration of SA modulations to activate plant defence. On the one hand, *OsNPR1* is involved in the SAR mechanism of plant resistance (Fu and Dong, 2013) and could mediate antagonistic crosstalk between the SA- and JA-dependent pathways in rice (Chern et al., 2005). On the other hand, *OsWRKY45* plays a pivotal role in the BTH-induced defence response in rice by acting through the SA signalling pathway (Shimono et al., 2007). In our study, transcripts of both *OsNPR1* and *OsWRKY45* were significantly accumulated in Zh11, for 1 or 2 days after *M. graminicola* infection, suggesting that both SA-mediated pathways could be involved in the resistance. Besides, SA-related *OsMAPK5a* and *OsWRKY45* were transiently induced at early stages of infection in Zh11. In rice, *OsMAPK5a* encodes a stress-responsive mitogen-activated protein kinase (MAPK) whose expression is inducible by SA exogenous treatment (Nanda et al., 2018) and altered following infection by PPNs during compatible interactions in *O. sativa* Japonica Nipponbare (Nguyễn et al., 2014). Interestingly, those two genes showed the same pattern of regulation in the *M. graminicola*-resistant *O. sativa* variety Vadana (Kumari et al., 2016). However, the mechanism of resistance that takes place in Vadana has not been established yet. By contrast, neither *OsMAPK5a* nor *OsWRKY45* were significantly upregulatd in the TOG5681 *O. glaberrima* rice variety upon *M. graminicola* infection (Petitot et al., 2017), but in that case there is no HR involved in resistance. Furthermore, in *R*-mediated response to both virulent and avirulent *M. oryzae* in rice, the expression of *OsMAPK5a* and *OsWRKY45* followed the same pattern as in *M. graminicola*-infected Zh11, with a larger fold change expression ratio for *OsWRKY45* than *OsMAPK5a* like in response to nematodes, and with a stronger induction in response to the avirulent strain, which triggers HR (Delteil et al., 2012). It might therefore be possible that those two genes represent a feature of HR-dependent resistance in rice when SA is involved.

Finally, the resistance provided by Zh11 appears to be controlled by a complex response where phytohormones provide transient and interconnected responses. The concerted role of the two hormones, JA and SA, has previously been observed in incompatible interactions in rice with *M. graminicola*, in resistant *O. glaberrima* background, but also with the rice stem nematode *Ditylenchus angustus* (Petitot et al., 2017; Khanam et al., 2018). Molecular genetic experiments and the application of exogenous hormones (Gheysen and Mitchum, 2019) should enable a more precise description of the involvement of SA/JA biosynthesis and/or signal transduction pathways in the Zh11 hypersensitive response to *M. graminicola* infection in future studies.

### Resistance to *Meloidogyne graminicola* in Zhonghua 11 Is Oligogenic With Most Likely Two Complementary Dominant Genes Involved

Using genome-wide association studies of global rice panels including wild rice accessions and cultivated rice varieties, several quantitative trait loci (QTL) associated with *M. graminicola* resistance in rice were identified (Shrestha et al., 2007; Dimkpa et al., 2016; Galeng-Lawilao et al., 2018; Galeng-Lawilao et al., 2019; Lahari et al., 2019; Hada et al., 2020). Depending on the study, the level of resistance was assessed based on either the gall index or the reproduction factor and the analyses suggested that *M. graminicola* resistance in rice is a multi-factorial trait. Resistance appears to involve a number of genes scattered across several chromosomes that regulate a cascade of defence responses in rice (Hada et al., 2020). Epistatic interactions between loci could be observed that complicate the study of the genetic determinism of resistance (Galeng-Lawilao et al., 2018). While chromosome 11 appears to harbour a major QTL for *M. graminicola* resistance related to root gall formation (Lahari et al., 2019), other QTLs have been found in almost all chromosomes. For example, QTLs associated with root gall were mapped on chromosomes 1, 3, 4, 5, 11, and 12 in a global Asian rice panel study comprising 332 diverse *O. sativa* varieties (Dimkpa et al., 2016), and 11 QTLs associated with the nematode reproduction factor were identified by a GWAS approach using 272 diverse wild rice accessions, including *O. nivara*, *O. rufipogon*, and *O. sativa* f. *spontanea* species (Hada et al., 2020). Genetic studies hence suggest that *M. graminicola* resistance in rice may depend on the presence and interaction of many genes. Therefore, breeding high-yielding and *M. graminicola*-resistant hybrid rice varieties may be a challenge. Here we investigated the nature of the resistance to *M. graminicola* in Zh11 and present its potential use as a resistance donor for breeding.

For the genetic study, a total of 432 F_2_ progenies from crosses between the resistant variety Zh11 and the susceptible varieties IR64 and Nipponbare were assessed for susceptibility to *M. graminicola* infection, based on the nematode reproduction factor. We used both indica × japonica and japonica × japonica crosses to investigate the complex genetic interaction between loci carrying resistance traits. Indeed, as mentioned above, rice resistance to *M. graminicola* has been shown to involve several loci and potentially the resistance genes could exhibit epistatic interactions (Galeng-Lawilao et al., 2018). Our analysis supports the existence of two major loci involved in resistance in Zh11 that can be introgressed in either *O. sativa* indica or *O. sativa* japonica. A preliminary study had already shown that the *M. graminicola* resistance in Zh11 was conferred by at least one dominant gene. Indeed, all F_1_ hybrids obtained from the inter-subspecific cross Zh11 × IR64 were resistant to *M. graminicola* (Phan et al., 2018). In the present study F_1_ hybrids from the japonica × japonica cross between Zh11 and Nipponbare were also resistant, thus confirming the presence of at least one dominant resistance gene. Moreover, we were able to study the segregation of the resistance in the F_2_ populations resulting from self-fertilisation of F_1_ individuals coming from either cross. The observed segregation ratios were 11R:5S and 9R:7S, depending on the analysed cross. The 9R:7S ratio observed in F_2_s from the Nipponbare × Zh11 intra-subspecific cross confirmed the existence of two unlinked, complementary genes, and suggested that the dominant allele for each of the two loci is essential to fully confer resistance to *M. graminicola* (Miko, 2008), as has been observed in another pathosystem (Fredua-Agyeman et al., 2020). The 11R:5S ratio observed in the progeny of the indica × japonica cross, IR64 × Zh11, could correspond to a 9:7 ratio too, only slightly altered by segregation distortion, which is a very common phenomenon in rice inter-subspecific crosses (Xian-Liang et al., 2006; Fragoso et al., 2017). The two independent crosses therefore point towards an oligogenic nature for the resistance carried by Zh11, with most likely two complementary dominant genes involved.

The finding that the resistance to *M. graminicola* involves at least two loci in *O. sativa* japonica Zh11 is not surprising and is consistent with the results of previous GWAS studies based on wide rice panels, which indicated that resistance to *M. graminicola* is supported by several QTLs (Dimkpa et al., 2016; Hada et al., 2020). Further bulked-segregant analyses of the progenies from the two resistant varieties LD24 and Khao Pahk Maw with a moderately susceptible cultivar, Vialone Nano (a temperate japonica from Italy), revealed that the introgression of the same locus is responsible for resistance in both cultivars, mapped to a 23-Mbp region at the bottom of rice chromosome 11, containing numerous NLR resistance genes (Lahari et al., 2019). Interestingly, when the 332 accessions of the rice diversity panel were assessed for gall formation in the original screening study that identified LD24 and Khao Pahk Maw as resistant to *M. graminicola*, it revealed a large variation across all subpopulations of rice and higher susceptibility in temperate japonica accessions (Dimkpa et al., 2016). We also noticed in our pathogenicity assays that Nipponbare is more susceptible than IR64 (Figure 1). We also found that some F_2_s from the inter-subspecific cross IR64 × Zh11 were transgressive, showing hypersensitivity to *M. graminicola* compared to IR64. This suggests that either minor *M. graminicola* resistance genes were already fixed in IR64 that have been lost by recombination in those F_2_s, which did not inherit the Zh11 resistance, or that these F_2_s express a set of so-called susceptibility genes that were previously absent in the IR64 background. In any case, even if many minor genes might be involved in the trait control, our study shows that the resistance to *M. graminicola* in Zh11 is highly heritable and is mainly controlled by two genes.

Resistance mediated by gene pairs has already been described in other plant-*Meloidogyne* pathosystems. For instance, in a carrot inbred line, two recessive genes regulate resistance to *Meloidogyne hapla* (Wang and Goldman, 1996), and in the common bean line PI165426, one dominant and one recessive gene (i.e., *Me2* and *me3*) condition resistance to *Meloidogyne incognita* at 26°C (Omwega and Roberts, 1992). Unfortunately, none of those genes have been cloned so far and the nature of the resistances is still unknown. Finally, if several known resistance NLR proteins work in conjunction with each other, recent findings revealed that many NLRs actually require other NLR proteins to function (Adachi et al., 2019), a feature that may explain the oligomeric nature of some resistances such as Zh11. In paired NLR systems, one of the receptor proteins is a sensor that recognises the pathogen while the coupled helper NLR is involved in initiating immune signalling. For example, the rice paired NLRs RG5/RG4 (RESISTANCE GENE ANALOGUE 5/4) function together as a single biochemical unit that forms an heterocomplex receptor to mediate resistance against the rice blast fungus *M. oryzae*, where RGA5 can recognise two unrelated effectors by direct binding (Cesari et al., 2013). These paired partners are genetically linked (Okuyama et al., 2011) but it is not always the case. Indeed, in Solanaceae the NLR-REQUIRED FOR CELL DEATH (NRC) helper receptors are required by a large number of sensor NLRs but unlike typical paired NLRs, NRCs are not always clustered with sensor NLRs in plant genomes (Wu et al., 2017). In Zh11 the two genes seemingly required for resistance are unlinked. Future mapping and identification of the gene loci underlying resistance to *M. graminicola* in Zh11 will tell us if it could belong to this type of paired network system, with important implications in term of breeding for resistance.

## Data Availability Statement

The original contributions presented in the study are included in the article/Supplementary Material, further inquiries can be directed to the corresponding author.

## Author Contributions

HN and SB conceived and designed the research that was coordinated by SB, with input from ML, SM, and JJ. HN, ML, and CM performed the experiments, with technical advice provided by CH. HN and ML analysed the data. HN, SM, and SB wrote the manuscript, with input from ML and JJ. All authors read and approved the final manuscript.

## Conflict of Interest

The authors declare that the research was conducted in the absence of any commercial or financial relationships that could be construed as a potential conflict of interest.

## Publisher’s Note

All claims expressed in this article are solely those of the authors and do not necessarily represent those of their affiliated organizations, or those of the publisher, the editors and the reviewers. Any product that may be evaluated in this article, or claim that may be made by its manufacturer, is not guaranteed or endorsed by the publisher.

## References

[B2] Adachi H. Derevnina L. Kamoun S. (2019). NLR singletons, pairs, and networks: evolution, assembly, and regulation of the intracellular immunoreceptor circuitry of plants. *Curr. Opin. Plant Biol.* 50 121–131. 10.1016/j.pbi.2019.04.007 31154077

[B3] Balint-Kurti P. (2019). The plant hypersensitive response: concepts, control and consequences. *Mol. Plant Pathol.* 20 1163–1178. 10.1111/MPP.12821 31305008 PMC6640183

[B4] Barbary A. Djian-Caporalino C. Palloix A. Castagnone-Sereno P. (2015). Host genetic resistance to root-knot nematodes, *Meloidogyne* spp., in Solanaceae: from genes to the field. *Pest Manag. Sci.* 71 1591–1598. 10.1002/ps.4091 26248710

[B5] Bellafiore S. Jougla C. Chapuis É Besnard G. Suong M. Vu P. N. (2015). Intraspecific variability of the facultative meiotic parthenogenetic root-knot nematode (*Meloidogyne graminicola*) from rice fields in Vietnam. *Comptes Rendus Biol.* 338 471–483. 10.1016/j.crvi.2015.04.002 26026576

[B6] Bellafiore S. Shen Z. Rosso M. N. Abad P. Shih P. Briggs S. P. (2008). Direct identification of the *Meloidogyne incognita* secretome reveals proteins with host cell reprogramming potential. *PLoS Pathog.* 4:e1000192. 10.1371/journal.ppat.1000192 18974830 PMC2568823

[B7] Bhattacharjee S. (2012). The language of reactive oxygen species signaling in plants. *J. Exp. Bot.* 2012:985298. 10.1155/2012/985298

[B8] Bimpong I. K. Carpena A. L. Mendioro M. S. Fernandez L. Ramos J. Reversat G. (2010). Evaluation of *Oryza sativa* x *O. glaberrima* derived progenies for resistance to root-knot nematode and identification of introgressed alien chromosome segments using SSR markers. *Afr. J. Biotechnol.* 9 3988–3997. 10.4314/ajb.v9i26

[B9] Branch C. Hwang C. F. Navarre D. A. Williamson V. M. (2004). Salicylic acid is part of the Mi-1-mediated defense response to root-knot nematode in tomato. *Mol. Plant Microbe Interact.* 17 351–356. 10.1094/mpmi.2004.17.4.351 15077667

[B10] Bridge J. Page S. L. J. (1982). The rice root-knot nematode, *Meloidogyne graminicola*, on deep water rice (*Oryza sativa* subsp. indica). *Rev. Nematol.* 5 225–232.

[B11] Cabasan M. T. N. Kumar A. Bellafiore S. De Waele D. (2014). Histopathology of the rice root-knot nematode, *Meloidogyne graminicola*, on *Oryza sativa* and *O. glaberrima*. *Nematology* 16 73–81. 10.1163/15685411-00002746

[B12] Cabasan M. T. N. Kumar A. De Waele D. (2012). Comparison of migration, penetration, development and reproduction of *Meloidogyne graminicola* on susceptible and resistant rice genotypes. *Nematology* 14 405–415. 10.1163/156854111x602613

[B13] Callahan H. L. Crouch R. K. James E. R. (1988). Helminth anti-oxidant enzymes: a protective mechanism against host oxidants? *Parasitol. Today* 4 218–225. 10.1016/0169-4758(88)90162-7 15463102

[B14] Cao J. Zhang M. Xiao J. Li X. Yuan M. Wang S. (2018). Dominant and recessive major R genes lead to different types of host cell death during resistance to *Xanthomonas oryzae* in rice. *Front. Plant Sci.* 871:1711. 10.3389/fpls.2018.01711 30519255 PMC6258818

[B15] Cao J. Zhang M. Zhu M. He L. Xiao J. Li X. (2019). Autophagy-like cell death regulates hydrogen peroxide and calcium ion distribution in Xa3/Xa26-mediated resistance to *Xanthomonas oryzae* pv. oryzae. *Int. J. Mol. Sci.* 21:194. 10.3390/ijms21010194 31892124 PMC6981989

[B16] Cesari S. Thilliez G. Ribot C. Chalvon V. Michel C. Jauneau A. (2013). The rice resistance protein pair RGA4/RGA5 recognizes the *Magnaporthe oryzae* effectors AVR-Pia and AVR1-CO39 by direct binding. *Plant Cell* 25 1463–1481. 10.1105/tpc.112.107201 23548743 PMC3663280

[B17] Chern M. Fitzgerald H. A. Canlas P. E. Navarre D. A. Ronald P. C. (2005). Overexpression of a rice NPR1 homolog leads to constitutive activation of defense response and hypersensitivity to light. *Mol. Plant Microbe Interact.* 18 511–520. 10.1094/mpmi-18-0511 15986920

[B18] Choi H. W. Klessig D. F. (2016). DAMPs, MAMPs, and NAMPs in plant innate immunity. *BMC Plant Biol.* 16:232. 10.1186/s12870-016-0921-2 27782807 PMC5080799

[B19] Claverie M. Dirlewanger E. Bosselut N. Van Ghelder C. Voisin R. Kleinhentz M. (2011). The Ma gene for complete-spectrum resistance to *Meloidogyne* species in Prunus is a TNL with a huge repeated C-terminal post-LRR region. *Plant Physiol.* 156 779–792. 10.1104/pp.111.176230 21482634 PMC3177275

[B20] Cohen S. P. Leach J. E. (2019). Abiotic and biotic stresses induce a core transcriptome response in rice. *Sci. Rep.* 9 1–11. 10.1038/s41598-019-42731-8 31000746 PMC6472405

[B21] Daudi A. O’Brien J. A. (2012). Detection of hydrogen peroxide by DAB staining in *Arabidopsis* leaves. *Bio Protoc.* 2:e263. 10.21769/BioProtoc.263 PMC4932902 27390754

[B22] Davies L. J. Elling A. A. (2015). Resistance genes against plant-parasitic nematodes: a durable control strategy? *Nematology* 17 249–263. 10.1163/15685411-00002877

[B23] De Vleesschauwer D. Xu J. Höfte M. (2014). Making sense of hormone-mediated defense networking: from rice to Arabidopsis. *Front. Plant Sci.* 5:611. 10.3389/fpls.2014.00611 25426127 PMC4227482

[B24] De Waele D. Elsen A. (2007). Challenges in tropical plant nematology. *Annu. Rev. Phytopathol.* 45 457–485. 10.1146/annurev.phyto.45.062806.094438 17489690

[B25] Delledonne M. Zeier J. Marocco A. Lamb C. (2001). Signal interactions between nitric oxide and reactive oxygen intermediates in the plant hypersensitive disease resistance response. *Proc. Natl. Acad. Sci. U.S.A.* 98 13454–13459. 10.1073/pnas.231178298 11606758 PMC60892

[B26] Delteil A. Blein M. Faivre-Rampant O. Guellim A. Estevan J. Hirsch J. (2012). Building a mutant resource for the study of disease resistance in rice reveals the pivotal role of several genes involved in defence. *Mol. Plant Pathol.* 13 72–82. 10.1111/j.1364-3703.2011.00731.x 21726398 PMC6638870

[B27] Dimkpa S. O. N. Lahari Z. Shrestha R. Douglas A. Gheysen G. Price A. H. (2016). A genome-wide association study of a global rice panel reveals resistance in *Oryza sativa* to root-knot nematodes. *J. Exp. Bot.* 67 1191–1200. 10.1093/jxb/erv470 26552884 PMC4753847

[B28] Ebone L. A. Kovaleski M. Deuner C. C. (2019). Nematicides: history, mode, and mechanism action. *Plant Sci. Today* 6 91–97. 10.14719/pst.2019.6.2.468

[B29] Fragoso C. A. Moreno M. Wang Z. Heffelfinger C. Arbelaez L. J. Aguirre J. A. (2017). Genetic architecture of a rice nested association mapping population. *G3 (Bethesda)* 7 1913–1926. 10.1534/g3.117.041608 28450374 PMC5473768

[B30] Fredua-Agyeman R. Jiang J. Hwang S. F. Strelkov S. E. (2020). QTL mapping and inheritance of clubroot resistance genes derived from *Brassica rapa* subsp. rapifera (ECD 02) reveals resistance loci and distorted segregation ratios in two F_2_ populations of different crosses. *Front. Plant Sci.* 11:899. 10.3389/fpls.2020.00899 32719696 PMC7348664

[B31] Fu Z. Q. Dong X. (2013). Systemic acquired resistance: turning local infection into global defense. *Annu. Rev. Plant Biol.* 64 839–863. 10.1146/annurev-arplant-042811-105606 23373699

[B32] Galeng-Lawilao J. Kumar A. De Waele D. (2018). QTL mapping for resistance to and tolerance for the rice root-knot nematode, *Meloidogyne graminicola*. *BMC Genet.* 19:53. 10.1186/s12863-018-0656-1 30081817 PMC6080554

[B33] Galeng-Lawilao J. G. Swamy B. P. M. Kumar A. Cabasan M. T. N. De Waele D. (2019). Mapping quantitative trait loci of *Meloidogyne graminicola* resistance and tolerance in a recombinant inbred line population of *Oryza glaberrima* × *O. sativa*. *Nematology* 21 401–417. 10.1163/15685411-00003222

[B34] Ganesan V. Thomas G. (2001). Salicylic acid response in rice: influence of salicylic acid on H_2_O_2_ accumulation and oxidative stress. *Plant Sci.* 160 1095–1106. 10.1016/s0168-9452(01)00327-2 11337066

[B35] Gartner U. Hein I. Brown L. H. Chen X. Mantelin S. Sharma S. K. (2021). Resisting potato cyst nematodes with resistance. *Front. Plant Sci.* 12:661194. 10.3389/fpls.2021.661194 33841485 PMC8027921

[B36] Gheysen G. Mitchum M. G. (2019). Phytoparasitic nematode control of plant hormone pathways. *Plant Physiol.* 179 1212–1226. 10.1104/pp.18.01067 30397024 PMC6446774

[B37] Gou W. Li X. Guo S. Liu Y. Li F. Xie Q. (2019). Autophagy in plant: a new orchestrator in the regulation of the phytohormones homeostasis. *Int. J. Mol. Sci.* 20:2900. 10.3390/ijms20122900 31197094 PMC6627538

[B38] Hada A. Dutta T. K. Singh N. Singh B. Rai V. Singh N. K. (2020). A genome-wide association study in Indian wild rice accessions for resistance to the root-knot nematode *Meloidogyne graminicola*. *PLoS One* 15:e0239085. 10.1371/journal.pone.0239085 32960916 PMC7508375

[B39] Hendy H. Dalmasso A. Cardin M. C. (1985). Differences in resistant *Capsicum annuum* attacked by different *Meloidogyne* species. *Nematologica* 31 72–78. 10.1163/187529285x00094

[B40] Henry S. Divol F. Bettemburg M. Bureau C. Guiderdoni E. Périn C. (2016). Immunoprofiling of rice root cortex reveals two cortical subdomains. *Front. Plant Sci.* 6:1139. 10.3389/fpls.2015.01139/bibtex 26779208 PMC4703777

[B41] Hoagland D. R. Arnon D. I. (1938). The water-culture method for growing plants without soil. *Circ. Calif. Agric. Exp. Station* 347 1–40. 10.54026/esecr/1046

[B42] Jena R. N. Rao Y. S. (1977). Nature of resistance in rice (*Oryza sativa* L) to the root-knot nematode (*Meloidogyne graminicola* Golden and Birchfield) II. Histopathology of nematode infection in rice varieties. *Proc. Indian Acad. Sci.* 86 87–91. 10.1007/bf03050910

[B43] Ji H. Gheysen G. Denil S. Lindsey K. Topping J. F. Nahar K. (2013). Transcriptional analysis through RNA sequencing of giant cells induced by *Meloidogyne graminicola* in rice roots. *J. Exp. Bot.* 64 3885–3898. 10.1093/jxb/ert219 23881398 PMC3745741

[B44] Kaloshian I. Teixeira M. (2019). Advances in plant-nematode interactions with emphasis on the notorious nematode genus *Meloidogyne*. *Phytopathology* 109 1988–1996. 10.1094/phyto-05-19-0163-ia 31613704

[B45] Khallouk S. Voisin R. Van Ghelder C. Engler G. Amiri S. Esmenjaud D. (2011). Histological mechanisms of the resistance conferred by the Ma gene against *Meloidogyne incognita* in *Prunus* spp. *Phytopathology* 101 945–951. 10.1094/phyto-01-11-0004 21446787

[B46] Khanam S. Bauters L. Singh R. R. Verbeek R. Haeck A. Sultan S. M. (2018). Mechanisms of resistance in the rice cultivar Manikpukha to the rice stem nematode *Ditylenchus angustus*. *Mol. Plant Pathol.* 19 1391–1402. 10.1111/mpp.12622 28990717 PMC6638125

[B47] Kim S. G. Kim S. T. Wang Y. Yu S. Choi I. S. Kim Y. C. (2011). The RNase activity of rice probenazole-induced protein1 (PBZ1) plays a key role in cell death in plants. *Mol. Cells* 3 25–31. 10.1007/s10059-011-0004-z 21110127 PMC3906867

[B48] Kumari C. Dutta T. K. Banakar P. Rao U. (2016). Comparing the defence-related gene expression changes upon root-knot nematode attack in susceptible versus resistant cultivars of rice. *Sci. Rep.* 6 1–13. 10.1038/srep22846 26961568 PMC4785349

[B49] Künstler A. Bacsó R. Gullner G. Hafez Y. M. Király L. (2016). Staying alive – is cell death dispensable for plant disease resistance during the hypersensitive response? *Physiol. Mol. Plant Pathol.* 93 75–84. 10.1016/j.pmpp.2016.01.003

[B50] Kyndt T. Nahar K. Haegeman A. De Vleesschauwer D. Höfte M. Gheysen G. (2012). Comparing systemic defence-related gene expression changes upon migratory and sedentary nematode attack in rice. *Plant Biol.* 14 73–82. 10.1111/j.1438-8677.2011.00524.x 22188265

[B51] Kyndt T. Vieira P. Gheysen G. de Almeida-Engler J. (2013). Nematode feeding sites: unique organs in plant roots. *Planta* 238 807–818. 10.1007/s00425-013-1923-z 23824525

[B52] Kyndt T. Zemene H. Y. Haeck A. Singh R. De Vleesschauwer D. Denil S. (2017). Below-ground attack by the root knot nematode *Meloidogyne graminicola* predisposes rice to blast disease. *Mol. Plant Microbe Interact.* 30 255–266. 10.1094/mpmi-11-16-0225-r 28151048

[B53] Lahari Z. Ribeiro A. Talukdar P. Martin B. Heidari Z. Gheysen G. (2019). QTL-seq reveals a major root-knot nematode resistance locus on chromosome 11 in rice (*Oryza sativa* L.). *Euphytica* 215 1–13. 10.1007/s10681-019-2427-0 31274875 PMC6570777

[B54] Leary A. Y. Sanguankiattichai N. Duggan C. Tumtas Y. Pandey P. Segretin M. E. (2018). Modulation of plant autophagy during pathogen attack. *J. Exp. Bot.* 69 1325–1333. 10.1093/jxb/erx425 29294077

[B55] Lee M.-W. Qi M. Yang Y. (2001). A novel jasmonic acid-inducible rice myb gene associates with fungal infection and host cell death. *Mol. Plant Microbe Interact.* 14 527–535. 10.1094/mpmi.2001.14.4.527 11310740

[B56] Liao C. Y. Bassham D. C. (2020). Combating stress: the interplay between hormone signaling and autophagy in plants. *J. Exp. Bot.* 71 1723–1733. 10.1093/jxb/erz515 31725881 PMC7067298

[B57] Livak K. J. Schmittgen T. D. (2001). Analysis of relative gene expression data using real-time quantitative PCR and the 2^−Δ^ ^Δ^ *^CT^* method. *Methods* 25 402–408. 10.1006/meth.2001.1262 11846609

[B58] Lorieux M. Reversat G. Garcia Diaz S. X. Denance C. Jouvenet N. Orieux Y. (2003). Linkage mapping of Hsa-1Og, a resistance gene of African rice to the cyst nematode, *Heterodera sacchari*. *Theor. Appl. Genet.* 107 691–696. 10.1007/s00122-003-1285-1 12721640

[B59] Mantelin S. Bellafiore S. Kyndt T. (2017). *Meloidogyne graminicola*: a major threat to rice agriculture. *Mol. Plant Pathol.* 18 3–15. 10.1111/mpp.12394 26950515 PMC6638252

[B60] Mattos V. S. Leite R. R. Cares J. E. Gomes A. C. M. M. Moita A. W. Lobo V. L. S. (2019). *Oryza glumaepatula*, a new source of resistance to *Meloidogyne graminicola* and histological characterization of its defense mechanisms. *Phytopathology* 109 1941–1948. 10.1094/phyto-02-19-0044-r 31215839

[B61] McClure M. A. M. Kruk T. H. Misaghi I. (1973). A method for obtaining quantities of clean *Meloidogyne* eggs. *J. Nematol.* 5:230. 19319340 PMC2620009

[B62] Melillo M. T. Leonetti P. Bongiovanni M. Castagnone-Sereno P. Bleve-Zacheo T. (2006). Modulation of reactive oxygen species activities and H_2_O_2_ accumulation during compatible and incompatible tomato–root-knot nematode interactions. *New Phytol.* 170 501–512. 10.1111/j.1469-8137.2006.01724.x 16626472

[B63] Miko I. (2008). Epistasis: gene interaction and phenotype effects. *Nature Educ.* 1:197. 10.1016/b978-0-12-375142-3.10012-4

[B64] Milligan S. B. Bodeau J. Yaghoobi J. Kaloshian I. Zabel P. Williamson V. M. (1998). The root knot nematode resistance gene Mi from tomato is a member of the leucine zipper, nucleotide binding, leucine-rich repeat family of plant genes. *Plant Cell* 10 1307–1319. 10.1105/tpc.10.8.1307 9707531 PMC144378

[B65] Nahar K. Kyndt T. De Vleesschauwer D. Höfte M. Gheysen G. (2011). The jasmonate pathway is a key player in systemically induced defense against root knot nematodes in rice. *Plant Physiol.* 157 305–316. 10.1104/pp.111.177576 21715672 PMC3165880

[B66] Nanda S. Wan P. J. Yuan S. Y. Lai F. X. Wang W. X. Fu Q. (2018). Differential responses of OsMPKs in IR56 rice to two BPH populations of different virulence levels. *Int. J. Mol. Sci.* 19:4030. 10.3390/ijms19124030 30551584 PMC6320944

[B67] Netscher C. Erlan. (1993). A root-knot nematode, *Meloidogyne graminicola*, parasitic on rice in Indonesia. *Afro Asia J. Nematol.* 3 90–95.

[B68] Nguyễn P. V. Bellafiore S. Petitot A.-S. Haidar R. Bak A. Abed A. (2014). *Meloidogyne incognita* – rice (*Oryza sativa*) interaction: a new model system to study plant-root-knot nematode interactions in monocotyledons. *Rice* 7:23. 10.1186/s12284-014-0023-4 26224554 PMC4884005

[B69] Nicol J. M. Turner S. J. Coyne D. L. Nijs L. Hockland S. Maafi Z. T. (2011). “Current nematode threats to world agriculture,” in *Genomics and Molecular Genetics of Plant-Nematode Interactions*, eds Jones J. Gheysen G. Fenoll C. (Dordrecht: Springer), 21–43. 10.1007/978-94-007-0434-3_2

[B70] OECD/FAO (2020). *“OECD-FAO Agricultural Outlook 2020-2029”, Agriculture Statistics Data Base.* Available online at: https://stats.oecd.org/viewhtml.aspx?datasetcode=HIGH_AGLINK_2020&lang=en (accessed December 14, 2021).

[B71] Okuyama Y. Kanzaki H. Abe A. Yoshida K. Tamiru M. Saitoh H. (2011). A multifaceted genomics approach allows the isolation of the rice Pia-blast resistance gene consisting of two adjacent NBS-LRR protein genes. *Plant J.* 66 467–479. 10.1111/j.1365-313X.2011.04502.x 21251109

[B72] Omwega C. O. Roberts P. A. (1992). Inheritance of resistance to *Meloidogyne* spp. in common bean and the genetic basis of its sensitivity to temperature. *Theor. Appl. Genet.* 83 720–726. 10.1007/bf00226690 24202746

[B73] Pegard A. Brizzard G. Fazari A. Soucaze O. Abad P. Djian-Caporalino C. (2005). Histological characterization of resistance to different root-knot nematode species related to phenolics accumulation in *Capsicum annuum*. *Phytopathology* 95 158–165. 10.1094/phyto-95-0158 18943985

[B74] Petitot A.-S. Dereeper A. Da Silva C. Guy J. Fernandez D. (2020). Analyses of the root-knot nematode (*Meloidogyne graminicola*) transcriptome during host infection highlight specific gene expression profiling in resistant rice plants. *Pathogens* 9:644. 10.3390/pathogens9080644 32784493 PMC7460394

[B75] Petitot A.-S. Kyndt T. Haidar R. Dereeper A. Collin M. de Almeida Engler J. (2017). Transcriptomic and histological responses of African rice (*Oryza glaberrima*) to *Meloidogyne graminicola* provide new insights into root-knot nematode resistance in monocots. *Ann. Bot.* 119 885–899. 10.1093/aob/mcw256 28334204 PMC5604615

[B76] Phan N. T. De Waele D. Lorieux M. Xiong L. Bellafiore S. (2018). A hypersensitivity-like response to *Meloidogyne graminicola* in rice (*Oryza sativa*). *Phytopathology* 108 521–528. 10.1094/phyto-07-17-0235-r 29161206

[B77] Plowright R. A. Coyne D. L. Nash P. Jones M. P. (1999). Resistance to the rice nematodes *Heterodera sacchari*, *Meloidogyne graminicola* and *M. incognita* in *Oryza glaberrima* and *O. glaberrima* x *O. sativa* interspecific hybrids. *Nematology* 1 745–751. 10.1163/156854199508775

[B78] Redeker K. R. Wang N. Y. Low J. C. McMillan A. Tyler S. C. Cicerone R. J. (2000). Emissions of methyl halides and methane from rice paddies. *Science* 290 966–969. 10.1126/science.290.5493.966 11062125

[B79] Reversat G. Fernandez L. (2004). Effect of inoculations with single and multiple juveniles on release of progeny of *Meloidogyne graminicola* from susceptible rice. *Nematology* 6 1–6. 10.1163/156854104323072856

[B80] Rich J. R. Brito J. A. Kaur R. Ferrell J. A. (2009). Weed species as hosts of *Meloidogyne*: a review. *Nematropica* 39 157–185.

[B81] Roy A. K. (1982). Survival of *Meloidogyne graminicola* eggs under different moisture conditions in vitro. *Nematol. Mediterr.* 10 221–222.

[B82] Sato K. Kadota Y. Shirasu K. (2019). Plant immune responses to parasitic nematodes. *Front. Plant Sci.* 10:1165. 10.3389/fpls.2019.01165 31616453 PMC6775239

[B83] Shimono M. Sugano S. Nakayama A. Jiang C. J. Ono K. Toki S. (2007). Rice WRKY45 plays a crucial role in benzothiadiazole-inducible blast resistance. *Plant Cell* 19 2064–2076. 10.1105/tpc.106.046250 17601827 PMC1955718

[B84] Shrestha R. Uzzo F. Wilson M. J. Price A. H. (2007). Physiological and genetic mapping study of tolerance to root-knot nematode in rice. *New Phytol.* 176 665–672. 10.1111/j.1469-8137.2007.02185.x 17822410

[B85] Shukla N. Yadav R. Kaur P. Rasmussen S. Goel S. Agarwal M. (2018). Transcriptome analysis of root-knot nematode (*Meloidogyne incognita*)-infected tomato (*Solanum lycopersicum*) roots reveals complex gene expression profiles and metabolic networks of both host and nematode during susceptible and resistance responses. *Mol. Plant Pathol.* 19 615–633. 10.1111/mpp.12547 28220591 PMC6638136

[B86] Soriano I. R. S. Prot J. C. Matias D. M. (2000). Expression of tolerance for *Meloidogyne graminicola* in rice cultivars as affected by soil type and flooding. *J. Nematol.* 32:309. 19270982 PMC2620461

[B87] Soriano I. R. Reversat G. (2003). Management of *Meloidogyne graminicola* and yield of upland rice in South-Luzon, Philippines. *Nematology* 5 879–884. 10.1163/156854103773040781

[B88] Soriano I. R. Schmit V. Brar D. S. Prot J.-C. Reversat G. (1999). Resistance to rice root-knot nematode *Meloidogyne graminicola* identified in *Oryza longistaminata* and *O. glaberrima*. *Nematology* 1 395–398. 10.1163/156854199508397

[B89] Takatsuji H. Jiang C. J. Sugano S. (2010). Salicylic acid signaling pathway in rice and the potential applications of its regulators. *Jpn. Agric. Res. Q.* 44 217–223. 10.6090/jarq.44.217

[B90] Thaler J. S. Humphrey P. T. Whiteman N. K. (2012). Evolution of jasmonate and salicylate signal crosstalk. *Trends Plant Sci.* 17 260–270. 10.1016/j.tplants.2012.02.010 22498450

[B91] Tian Z. Wang Z. Maria M. Qu N. Zheng J. (2019). *Meloidogyne graminicola* protein disulfide isomerase may be a nematode effector and is involved in protection against oxidative damage. *Sci. Rep.* 9:11949. 10.1038/s41598-019-48474-w 31420562 PMC6697734

[B92] Tian Z. Wang Z. Munawar M. Zheng J. (2020). Identification and characterization of a novel protein disulfide isomerase gene (MgPDI2) from *Meloidogyne graminicola*. *Int. J. Mol. Sci.* 21:9586. 10.3390/ijms21249586 33339262 PMC7767112

[B93] Ueno Y. Yoshida R. Kishi-Kaboshi M. Matsushita A. Jiang C. J. Goto S. (2015). Abiotic stresses antagonize the rice defence pathway through the tyrosine-dephosphorylation of OsMPK6. *PLoS Pathog.* 11:e1005231. 10.1371/journal.ppat.1005231 26485146 PMC4617645

[B94] van Wersch S. Tian L. Hoy R. Li X. (2020). Plant NLRs: the whistleblowers of plant immunity. *Plant Commun.* 1:100016. 10.1016/j.xplc.2019.100016 33404540 PMC7747998

[B95] Wang M. Goldman I. L. (1996). Resistance to root knot nematode (*Meloidogyne hapla* Chitwood) in carrot is controlled by two recessive genes. *J. Hered.* 87 119–123. 10.1093/oxfordjournals.jhered.a022966

[B96] Wang P. Wang T. Han J. Li M. Zhao Y. Su T. (2021). Plant autophagy: an intricate process controlled by various signaling pathways. *Front. Plant Sci.* 12:754982. 10.3389/fpls.2021.754982 34630498 PMC8495024

[B97] Wang Y. Wu J. Kim S. G. Tsuda K. Gupta R. Park S. Y. (2016). *Magnaporthe oryzae*-secreted protein MSP1 induces cell death and elicits defense responses in rice. *Mol. Plan Microbe Interact.* 29 299–312. 10.1094/mpmi-12-15-0266-r 26780420

[B98] Win P. P. Kyi P. P. Maung Z. T. Z. Myint Y. Y. Cabasan M. T. N. De Waele D. (2019). Crop rotation sequencing to minimize yield losses of summer-irrigated lowland rice in Myanmar caused by the rice root-knot nematode *Meloidogyne graminicola*. *Int. J. Pest Manag.* 66 319–331. 10.1080/09670874.2019.1647369

[B99] Wu C. H. Abd-El-Haliem A. Bozkurt T. O. Belhaj K. Terauchi R. Vossen J. H. (2017). NLR network mediates immunity to diverse plant pathogens. *Proc. Natl. Acad. Sci. U.S.A.* 114 8113–8118. 10.1073/pnas.1702041114 28698366 PMC5544293

[B100] Xian-Liang S. Xue-Zhen S. Tian-Zhen Z. (2006). Segregation distortion and its effect on genetic mapping in plants. *Chin. J. Agric. Biotechnol.* 3 163–169. 10.1079/CJB2006110

[B101] Xu G. Wang S. Han S. Xie K. Wang Y. Li J. (2017). Plant Bax Inhibitor-1 interacts with ATG6 to regulate autophagy and programmed cell death. *Autophagy* 13 1161–1175. 10.1080/15548627.2017.1320633 28537463 PMC5529081

[B102] Yamada S. Kano A. Tamaoki D. Miyamoto A. Shishido H. Miyoshi S. (2012). Involvement of OsJAZ8 in jasmonate-induced resistance to bacterial blight in rice. *Plant Cell Physiol.* 53 2060–2072. 10.1093/pcp/pcs145 23104764

[B103] Yang J. Duan G. Li C. Liu L. Han G. Zhang Y. (2019). The crosstalks between jasmonic acid and other plant hormone signaling highlight the involvement of jasmonic acid as a core component in plant response to biotic and abiotic stresses. *Front. Plant Sci.* 10:1349. 10.3389/fpls.2019.01349/bibtex 31681397 PMC6813250

[B104] Yang Y. Qi M. Mei C. (2014). Endogenous salicylic acid protects rice plants from oxidative damage caused by aging as well as biotic and abiotic stress. *Plant J.* 46 909–919. 10.1111/j.1365-313X.2004.02267.x 15584956

[B105] Zeng H. Y. Zheng P. Wang L. Y. Bao H. N. Sahu S. K. Yao N. (2019). Autophagy in plant immunity. *Adv. Exp. Med. Biol.* 1209 23–41. 10.1007/978-981-15-0606-2_3 31728863

[B106] Zhan L. Ding Z. Peng D. Peng H. Kong L. Liu S. (2018). Evaluation of Chinese rice varieties resistant to the root-knot nematode *Meloidogyne graminicola*. *J. Integr. Agric.* 17 621–630. 10.1016/s2095-3119(17)61802-1

