# Supplementary material for: Insights Into the Genetics of the Zhonghua 11 Resistance to Meloidogyne graminicola and Its Molecular Determinism in Rice
Source: Front Plant Sci. 2022 May 4;13:854961. doi: 10.3389/fpls.2022.854961 (PMC9116194; doi:10.3389/fpls.2022.854961)
Supplement: Supplementary file 1 [file Table_1.DOCX]

**Supporting table**

| **Gene name** | **RAP DB^(*)^ Gene Loci** | **Function** | **Forward primer (5’-3’)** | **Reverse primer (5’-3’)** | **References** |
| --- | --- | --- | --- | --- | --- |
| *OseEF-1A* | Os03g0177400 | Reference gene | GAAGTCTCATCCTACCTGAAGAAG | GTCAAGAGCCTCAAGCAAGG | Nguyễn *et al.* (2014) |
| *OsPAL4* | Os02g0627100 | SA-biosynthesis | CCTCGCCATCGCTGCCATC | GCCGTTGTTGTAGAAGTCGTTCAC | Petitot *et al.* (2017) |
| *OsMAPK20* | Os01g0629900 | SA signalling | TCAACTCCAATTCCTGCCAAG | AACAACTCTTCCTGGTCTTGC | Nguyễn *et al.* (2014) |
| *OsMAPK5a* | Os03g0285800 | SA-signalling | GTCTGCTCCGTGATGAAC | TGATGCCTATGATGTTCTCG | Nguyễn *et al.* (2014) |
| *OsWRKY45* | Os05g0322900 | SA-response | ACGACGAGGTTGTCTTCGATCTG | GCCCGTGTCCATCCATGATTCTTC | Petitot *et al.* (2017) |
| *OsNPR1* | Os01g0194300 | SA-response | AGAAGTCATTGCCTCCAG | ACATCGTCAGAGTCAAGG | Kumari *et al.* (2016) |
| *OsPR5* | Os12g0628600 | SA-response | CGCTGCCCCGACGCTTAC | ACGACTTGGTAGTTGCTGTTGC | Delteil *et al.* (2012) |
| *OsPR10a/PBZ1* | Os12g0555500 | SA-response | ACGCCTAAGATGAAGAGGAATAC | CTCAAACGCCACGAGAATTTG | Kumari *et al.* (2016) |
| *OsLOX4* | Os03g0700400 | JA-biosynthesis | TGGTGGAGCAGATCTACGTG | ATCGCCTTGATCGAGTAGCC | This study |
| *OsAOC* | Os03g0438100 | JA-biosynthesis | AAGAGGAATCGAGGACAAGATATTTG | AAGCCTCTTCTTGTTCGGATCA | This study |
| *OsPAD4* | Os11g0195500 | JA-signalling | TCAGAGGCAAGGCAGTAGTG | ACCGCTCACGCAGGATAG | Nguyễn *et al*. (2014) |
| *OsJAZ8* | Os09g0439200 | JA-signalling | GATGGCGTCGACAAGAACAC | TGCATTCATCCTGCCCTTTC | This study |
| *OsJAMYb* | Os11g0684000 | JA-response | GAGGACCAGAGTCAAAAGC | CATGGCATCCTTGAACCTCT | Nahar *et al*. (2011) |
| *OsJiOPR10* | Os03g0300400 | JA-response | CGGACGCTTACAACTAAATCG | AAACAAAACCATTCTCCGACAG | Nahar *et al*. (2011) |
| *OsAtg4* | Os04g0682000 | Autophagy | AGCACTGTACGAGATTTGGC | GTGGTGCTCCATTGGCTTTATC | This study |
| *OsAtg7* | Os01g0614900 | Autophagy | TCGAGCAATTGTACCGCATG | AGGCCGCTGATTTCATCAAG | This study |
| *OsBI-1* | Os02g0125300 | Autophagy | TACATCAAGCACGCACTCAC | ACGCGTTCTTGAGCATGATG | This study |

Table S1. **Primers used for gene expression studies.**

^(*)^ The gene loci provided are based on the Rice Annotation Project (RAP) data base (DB), available at <https://rapdb.dna.affrc.go.jp/>.
