# Supplementary material for: Insights Into the Genetics of the Zhonghua 11 Resistance to Meloidogyne graminicola and Its Molecular Determinism in Rice
Source: Front Plant Sci. 2022 May 4;13:854961. doi: 10.3389/fpls.2022.854961 (PMC9116194; doi:10.3389/fpls.2022.854961)
Supplement: Supplementary file 2 [file Table_2.docx]

**Supporting table**

**Table S2.** Chi-square test analysis of the segregation ratios for resistance to *Meloidogyne graminicola* in F_2_ populations from IR64 x Zh11 crosses based on the full dataset and when excluding from the dataset individual samples with Rf values falling in the ±5% or 10% interval around the threshold value that defines resistance.

| **IR64 x Zh11** | **Tested ratio** | **DF** | **Considering the full dataset** | | | | **Excluding values at threshold ±5%** | | | | **Excluding values at threshold ±10 %** | | | |
| --- | --- | --- | --- | --- | --- | --- | --- | --- | --- | --- | --- | --- | --- | --- |
|  |  |  | **Observed** | | **Test of statistics** | | **Observed** | | **Test of statistics** | | **Observed** | **Test of statistics** | | |
|  |  |  | **R (Rf ≤ 0.64)** | **S (Rf > 0.64)** | **X^2^** | ***p* value.** | **R (Rf ≤ 0.608)** | **S (Rf > 0.672)** | **X^2^** | ***p* value.** | **R (Rf ≤ 0.576)** | **S (Rf > 0.704)** | **X^2^** | ***p* value.** |
|  |  |  | 154 | 88 |  |  | 154 | 88 |  |  | 153 | 88 |  |  |
|  | 3:1 | 1 |  |  | 16.67 | 0.00006 |  |  | 16.67 | 0.00006 |  |  | 17.048 | 0.00004 |
|  | 1:3 | 1 |  |  | 192.67 | <0.00001 |  |  | 192.67 | <0.00001 |  |  | 190.371 | <0.00001 |
|  | 9:7 | 1 |  |  | 5.37 | 0.03 |  |  | 5.37 | 0.03 |  |  | 5.13 | 0.024 |
|  | 7:9 | 1 |  |  | 38.89 | <0.00001 |  |  | 38.89 | <0.00001 |  |  | 38.14 | <0.00001 |
|  | 5:11 | 1 |  |  | 118.15 | <0.00001 |  |  | 118.15 | <0.00001 |  |  | 116.56 | <0.00001 |
|  | **11:5** | **1** |  |  | **2.95** | **0.089** |  |  | **2.95** | **0.089** |  |  | **3.11** | **0.078** |
|  | 13:3 | 1 |  |  | 49.28 | <0.00001 |  |  | 49.28 | <0.00001 |  |  | 49.92 | <0.00001 |
|  | 3:13 | 1 |  |  | 320.05 | <0.00001 |  |  | 320.05 | <0.00001 |  |  | 316.59 | <0.00001 |
|  | 15:1 | 1 |  |  | 374.53 | <0.00001 |  |  | 374.53 | <0.00001 |  |  | 376.731 | <0.00001 |
|  | 1:15 | 1 |  |  | 1,360.13 | <0.00001 |  |  | 1,360.13 | <0.00001 |  |  | 1,347.40 | <0.00001 |
|  | 63:1 | 1 |  |  | 1,905.56 | <0.00001 |  |  | 1,905.56 | <0.00001 |  |  | 1,914.17 | <0.00001 |
|  | 1:63 | 1 |  |  | 6,062.51 | <0.00001 |  |  | 6,062.51 | <0.00001 |  |  | 6,008.14 | <0.00001 |
