# Supplementary material for: Insights Into the Genetics of the Zhonghua 11 Resistance to Meloidogyne graminicola and Its Molecular Determinism in Rice
Source: Front Plant Sci. 2022 May 4;13:854961. doi: 10.3389/fpls.2022.854961 (PMC9116194; doi:10.3389/fpls.2022.854961)
Supplement: Supplementary file 3 [file Table_3.docx]

**Supporting table**

**Table S3.** Chi-square test analysis of the segregation ratios for resistance to *Meloidogyne graminicola* in F_2_ populations from Nipponbare x Zh11 crosses based on the full dataset and when excluding from the dataset individual samples with Rf values falling in the ±5% or 10% interval around the threshold value that defines resistance.

| **Nipponbare x Zh11** | **Tested ratio** | **DF** | **Considering the full dataset** | | | | **Excluding values at threshold ±5%** | | | | **Excluding values at threshold ±10%** | | | |
| --- | --- | --- | --- | --- | --- | --- | --- | --- | --- | --- | --- | --- | --- | --- |
|  |  |  | **Observed** | | **Test of statistics** | | **Observed** | | **Test of statistics** | | **Observed** | | **Test of statistics** | |
|  |  |  | **R (Rf ≤ 2.5)** | **S (Rf >2.5)** | **X^2^** | ***p* value** | **R (Rf ≤ 2.45)** | **S (Rf >4.95)** | **X^2^** | ***p* value.** | **R (Rf ≤ 2.25)** | **S (Rf >4.75)** | **X^2^** | ***p* value.** |
|  |  |  | 98 | 81 |  |  | 97 | 67 |  |  | 97 | 67 |  |  |
|  | 3:1 | 1 |  |  | 39.15 | <0.00001 |  |  | 21.98 | <0.00001 |  |  | 21.98 | <0.00001 |
|  | 1:3 | 1 |  |  | 84.49 | <0.00001 |  |  | 101.98 | <0.00001 |  |  | 101.98 | <0.00001 |
|  | **9:7** | **1** |  |  | **0.16** | **0.7** |  |  | **0.56** | **0.4** |  |  | **0.56** | **0.4** |
|  | 7:9 | 1 |  |  | 8.80 | 0.0033 |  |  | 15.80 | 0.00007 |  |  | 15.80 | 0.00007 |
|  | 5:11 | 1 |  |  | 46.01 | <0.00001 |  |  | 59.40 | <0.0001 |  |  | 59.40 | <0.0001 |
|  | 11:5 | 1 |  |  | 16.33 | 0.00004 |  |  | 7.04 | 0.007 |  |  | 7.04 | 0.007 |
|  | 13:3 | 1 |  |  | 82.52 | <0.00001 |  |  | 52.60 | <0.00001 |  |  | 52.60 | <0.00001 |
|  | 3:13 | 1 |  |  | 152.26 | <0.00001 |  |  | 175.67 | <0.00001 |  |  | 175.67 | <0.00001 |
|  | 15:1 | 1 |  |  | 464.69 | <0.00001 |  |  | 335.15 | <0.00001 |  |  | 335.15 | <0.00001 |
|  | 1:15 | 1 |  |  | 718.56 | <0.00001 |  |  | 783.15 | <0.00001 |  |  | 783.15 | <0.00001 |
|  | 63:1 | 1 |  |  | 2,221.34 | <0.00001 |  |  | 1,646.09 | <0.00001 |  |  | 1,646.09 | <0.00001 |
|  | 1:63 | 1 |  |  | 3,292.07 | <0.00001 |  |  | 3,535.61 | <0.00001 |  |  | 3,535.61 | <0.00001 |
